# Dissecting key regulators of transcriptome kinetics through scalable single-cell RNA profiling of pooled CRISPR screens

**DOI:** 10.1038/s41587-023-01948-9

**Published:** 2023-09-25

**Authors:** Zihan Xu, Andras Sziraki, Jasper Lee, Wei Zhou, Junyue Cao

**Affiliations:** 1 Laboratory of Single Cell Genomics and Population Dynamics, The Rockefeller University, New York, NY USA; 2 The David Rockefeller Graduate Program in Bioscience, The Rockefeller University, New York, NY USA

**Keywords:** Transcriptomics, Gene regulation, Transcriptomics

## Abstract

We present a combinatorial indexing method, *PerturbSci-Kinetics*, for capturing whole transcriptomes, nascent transcriptomes and single guide RNA (sgRNA) identities across hundreds of genetic perturbations at the single-cell level. Profiling a pooled CRISPR screen targeting various biological processes, we show the gene expression regulation during RNA synthesis, processing and degradation, miRNA biogenesis and mitochondrial mRNA processing, systematically decoding the genome-wide regulatory network that underlies RNA temporal dynamics at scale.

## Abstract (web summary)

mRNA kinetics are described by combining single-cell combinatorial indexing with metabolic labeling and pooled CRISPR screens.

## Main

Cellular functions are determined by the expression of millions of RNA molecules, which are tightly regulated by their synthesis, splicing and degradation. However, understanding how key regulators impact genome-wide RNA kinetics is constrained by existing tools, which provide only snapshots of the transcriptome^1–8^. To resolve this challenge, we developed *PerturbSci-Kinetics*, combining CRISPR-based pooled genetic screen, single-cell RNA sequencing (RNA-seq) by combinatorial indexing and RNA metabolic labeling to uncover single-cell transcriptome dynamics across extensive genetic perturbations.

**Fig. 1:**
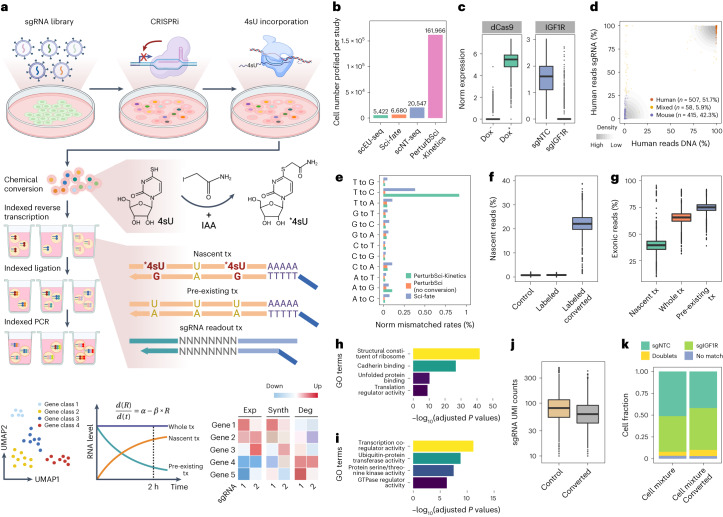
*PerturbSci-Kinetics* enables joint profiling of transcriptome dynamics and high-throughput gene perturbations. **a**, Scheme of *PerturbSci-Kinetics*. *4sU, chemically modified 4sU; *α* and Synth, RNA synthesis rate; *β* and Deg, RNA degradation rate; *R* and Exp, steady-state expression; tx, transcripts. **b**, Bar plot showing the cell numbers profiled in this study and those from published single-cell RNA-seq coupled with metabolic labeling^20–22^. **c**, Left, the log-transformed normalized expression of *dCas9-KRAB-MeCP2* in untreated (*n* = 3,344 cells) or dox-induced (*n* = 1,419 cells) HEK293-idCas9 cells. Right, the normalized expression of *IGF1R* in dox-induced HEK293-idCas9 cells transduced with sgNTC (*n* = 688 cells) or sgIGF1R (*n* = 820 cells). Norm, normalized. **d**, An equal number of induced HEK293-idCas9-sgIGF1R cells and 3T3-CRISPRi-sgFto cells were mixed and were profiled using *PerturbSci*. Scatter plot showed the concordance between percentage of transcriptome and sgRNA reads mapping to human and mouse genomes and human and mouse sgRNA, respectively, for each cell. **e**, Bar plot showing the sequencing-depth-normalized percentages of single-base mismatches in reads from sci-fate^20^ and *PerturbSci-Kinetics* on chemically converted or unconverted cells. **f**, Box plot showing the fraction of nascent reads recovered from single cells without 4sU labeling and chemical conversion (*n* = 1,498 cells), 4sU-labeled cells without chemical conversion (*n* = 1,008 cells) and 4sU-labeled/converted cells (*n* = 2,568 cells). **g**, Box plot showing the proportion of nascent, pre-existing and whole-transcriptome reads mapped to exons of the genome across single cells (*n* = 4,115 cells). **h**,**i**, Bar plots showing the enriched GO terms in genes with low (**h**) or high (**i**) nascent reads fractions. One-sided Fisher’s exact tests were conducted with the alternative hypothesis that the true odds ratio is greater than 1. **j**, Box plot showing the sgRNA UMI counts per cell in cells with (*n* = 2,568 cells) or without (*n* = 2,506 cells) the chemical conversion. **k**, Stacked bar plot showing the fraction of converted/unconverted cells identified as sgNTC/sgIGF1R singlets, doublets and cells with no sgRNA detected. Boxes in box plots indicate the median and interquartile range (IQR), with whiskers indicating 1.5× IQR.

**Extended Data Fig. 1:**
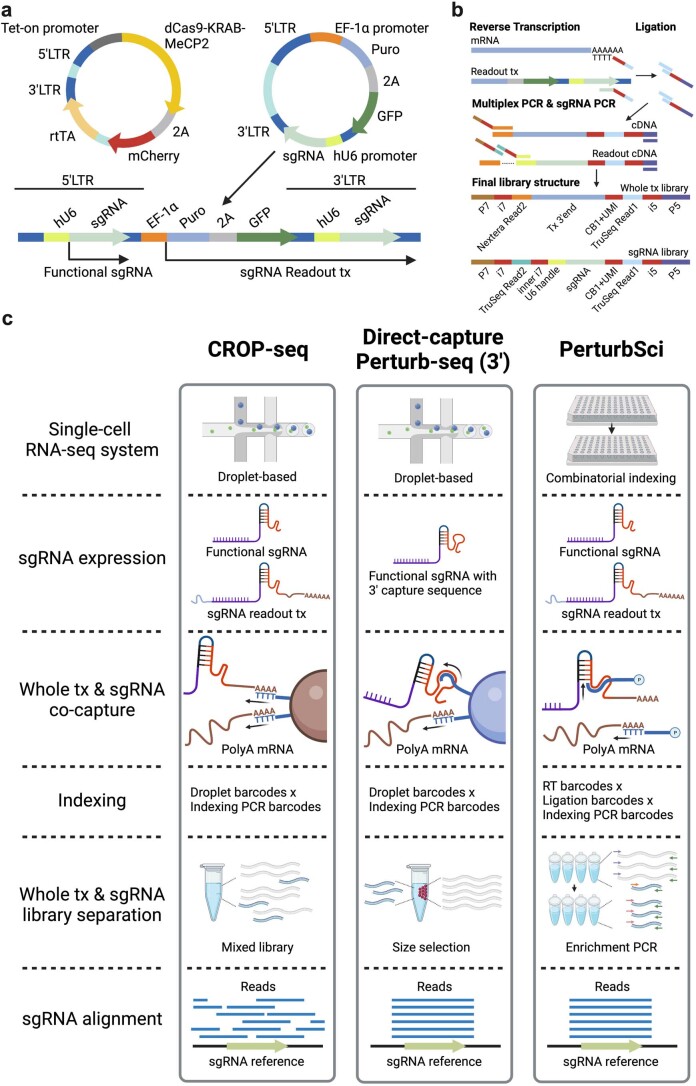
Scheme of plasmids and experiment procedures of *PerturbSci*. **a**. The vector system used in *PerturbSci* for dCas9 and sgRNA expression. The expression of the enhanced CRISPRi silencer dCas9-KRAB-MeCP2 (ref. ^9^) was controlled by the tetracycline responsive (Tet-on) promoter. A GFP sequence was added to the original CROP-seq-opti plasmid^6^ as an indicator of successful sgRNA transduction and for the lentivirus titer measurement. The CROP-seq vector utilizes the self-replication mechanism of lentivirus during the integration for amplifying the sgRNA expression cassette. In this lentiviral plasmid, the sgRNA expression cassette replaced the U3 region of the 3′LTR^5^. During the lentiviral integration, the shortened 5′LTR of reverse-transcribed lentiviral genomic DNA was extended by using its 3′LTR as a template, and the sgRNA expression cassette is self-replicated to the 5′LTR^62^. The self-replicated sgRNA expression cassette at 5′LTR generates functional sgRNA for guiding dCas9, and the original expression cassette at 3′LTR is transcribed as a part of the Puro-GFP fusion transcript driven by the EF-1α promoter. **b**. The library preparation scheme and the final library structures of *PerturbSci*, including a scalable combinatorial indexing strategy with direct sgRNA capture and enrichment that reduced the library preparation cost, enhanced the sensitivity of the sgRNA capture compared to the original CROP-seq^5^, and avoided the extensive barcodes swapping detected in Perturb-seq^6^. **c**. A schematic comparison of chemistries between *PerturbSci*, CROP-seq^5^, and Direct-capture Perturb-seq^7^.

**Extended Data Fig. 2:**
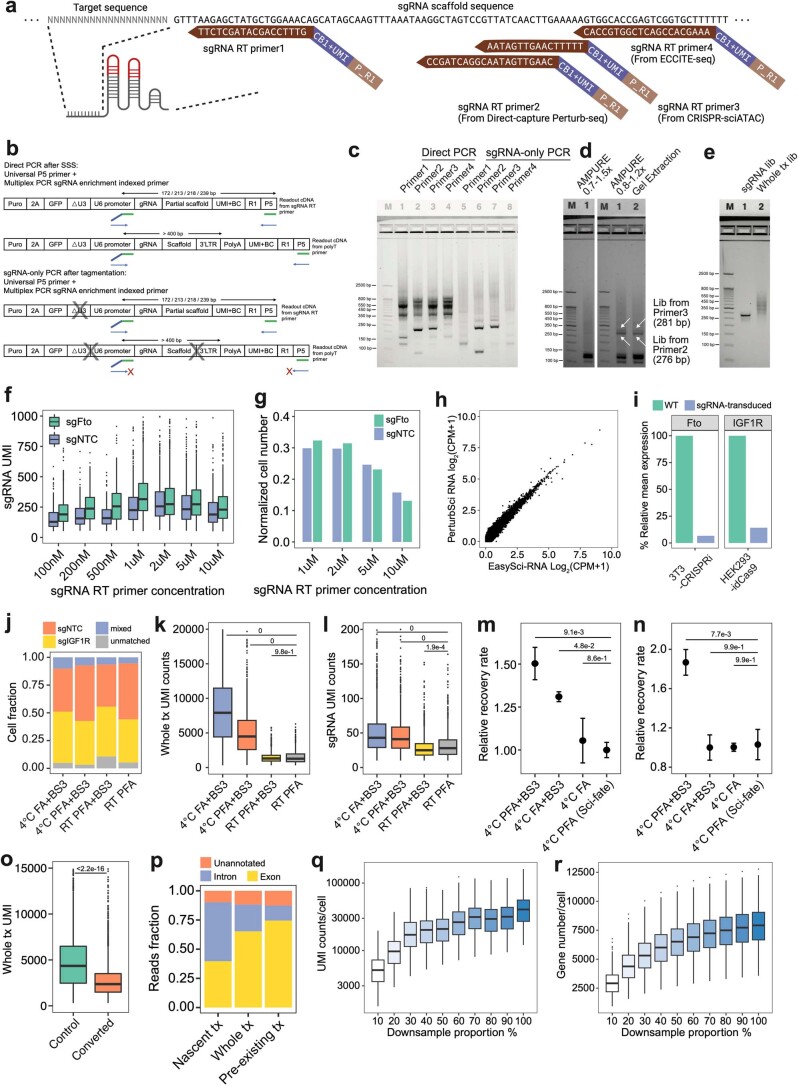
Representative optimizations of *PerturbSci*. **a**. sgRNA primers of different designs were mixed with polyT primers respectively for RT. CB, cell barcode. P_R1, partial TruSeq read1 sequence. **b-c**. After RT, the capture efficiency of sgRNA by different RT primers was evaluated by ‘Direct PCR’, and the efficiency of by-product removal was examined by ‘sgRNA-only PCR’. 3 independent experiments were conducted. **d**. Different post-multiplex PCR purification strategies were tested. 3 independent experiments were conducted. **e**. A representative gel image of libraries of *PerturbSci*. 5 independent experiments were conducted. **f-g**. Boxplots showing sgRNA UMI counts (f) and the cell number recovered (g) from different sgRNA primer concentrations (n = 230, 181, 149, 529, 512, 445, 299 cells from 100nM to 10uM groups for sgNTC cells, n = 499, 399, 246, 1237, 1215, 904, 537 cells from 100nM to 10uM groups for sgFto cells). **h**. Scatterplot showing the correlation between log2-transformed counts per million (CPM) profiled by *PerturbSci* and *EasySci*^10^ in the 3T3L1-CRISPRi cell line. **i**. Barplots showing effective knockdown in mouse 3T3-CRISPRi-sgFto cells and human HEK293-idCas9-sgIGF1R cells computationally assigned in the species-mixing experiment (Fig. 1d). **j–l**. Barplots showing the cell identities fraction (j), whole transcriptome (k) and sgRNA UMI counts (l) detected per cell in different fixation conditions (n = 1508, 1132, 1247, 1084 cells for conditions from the left to the right). Tukey’s tests after one-way ANOVA were performed. **m-n**. Dotplots showing the relative recovery rate (n = 4, mean ± SEM) of HEK293-idCas9 cells in different fixation conditions following HCl permeabilization (m) and chemical conversion (n). Dunnett’s test after one-way ANOVA was performed. **o**. Boxplot showing the effect of chemical conversion on whole transcriptome UMI counts under 4 °C PFA + BS3 fixation condition (n = 1988 cells in the control group, n = 4831 cells in the converted group). Two-sided Wilcoxon rank sum test was performed. **p**. Mapping statistics of reads from *PerturbSci-Kinetics*. **q-r**. Boxplots showing single-cell whole transcriptome UMI counts (q) and gene counts (r) under different sequencing depth (n = 500 cells in each subsampling group). Boxes in boxplots indicate the median and IQR with whiskers indicating 1.5× IQR. Source data

*PerturbSci-Kinetics* features a combinatorial indexing strategy (‘*PerturbSci*’) for targeted capture of single guide RNA (sgRNA) transcripts that carries the same cellular barcode with the whole transcriptome (Fig. 1a). In brief, we adopted the modified CRISPR droplet sequencing (CROP-seq) vector^5^ and developed a strategy for capturing sgRNA sequences^6,7^ through reverse transcription using an sgRNA-specific primer followed by targeted enrichment of sgRNA sequences via polymerase chain reaction (PCR) (Extended Data Fig. 1, Supplementary Notes 1 and 2 and Supplementary Table 1). With extensive optimizations (Extended Data Fig. 2), *PerturbSci* achieves a high knockdown efficacy with a potent dual-repressor dCas9 (that is, dCas9-KRAB-MeCP2; ref. ^9^) and a high capture rate of sgRNA (that is, up to 99.7% of cells) and can readily scale up for profiling a large number of cells using the three-level combinatorial indexing approach^10^ (Fig. 1b and Supplementary Note 3).

**Extended Data Fig. 3:**
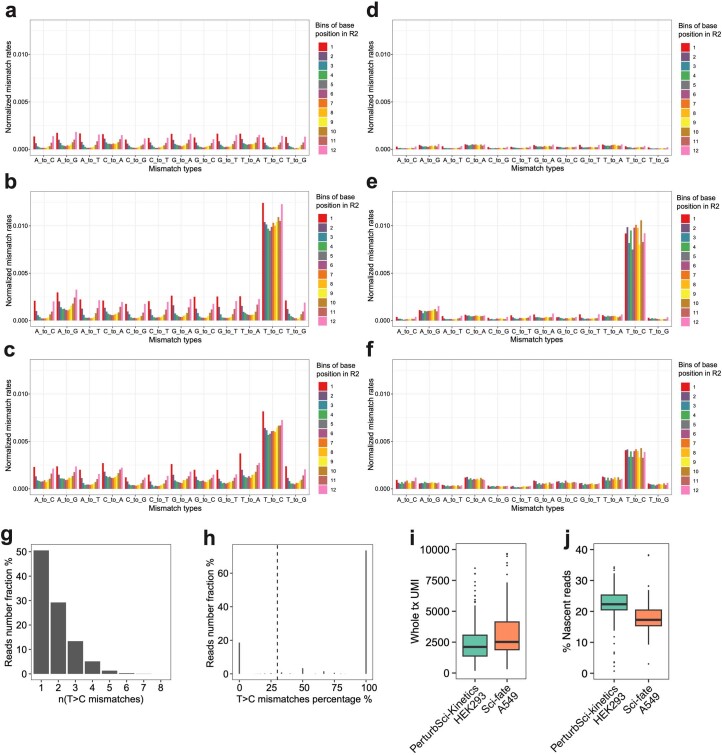
Optimization and benchmarking of the computational pipeline for nascent reads calling. **a–c**. Barplots showing the normalized mismatch rates of all 12 mismatch types detected in unconverted cells (a), converted cells (b), and the original sci-fate A549 dataset^20^ (c) at different positions of the reads using the original sci-fate mutation calling pipeline^20^. **d–f**. Barplots showing the normalized mismatch rates of all 12 mismatch types detected in unconverted cells (d), converted cells (e), and the original sci-fate A549 dataset^20^ (f) at different positions of the reads using the updated mutation calling pipeline. Considering the different sequencing lengths between the present dataset and sci-fate, the Read2 from sci-fate were trimmed to the same length as the present dataset before processing. Compared to the original pipeline, the updated pipeline further filtered the mismatch based on the CIGAR string and only mismatches with ‘CIGAR = M’ were kept. Normalized mismatch rates in each bin, the percentage of each type of mismatch in all sequencing bases within the bin. **g**, **h**. Statistics of T > C mutations in *PerturbSci-Kinetics* reads. Histogram showing the number of T > C mutations on reads that were identified to be from newly synthesized transcripts (g). For each read with high-quality mismatches identified, the fraction of mismatches from T > C mutations was calculated, which clearly separated the reads with background mutations and mutants introduced by 4sU in the plot (h). 30% was set as the cutoff to assign nascent reads as sci-fate^20^. **i**, **j**. Downsampling comparison between sci-fate^20^ and *PerturbSci-Kinetics*. A subset of raw reads in sci-fate A549 dataset^20^ were randomly selected to generate a downsampled dataset with the same single-cell raw reads number distribution with *PerturbSci-Kinetics*, and both datasets were processed using the same pipeline (n = 200 cells for each dataset). The single-cell whole transcriptome UMI counts (i) and the nascent reads proportions (j) between two datasets were compared. Boxes in boxplots indicate the median and IQR with whiskers indicating 1.5× IQR.

By incorporating 4-thiouridine (4sU) labeling^11–17^, *PerturbSci-Kinetics* retrieves time-resolved nascent transcriptomes at single-cell resolution, distinguishing newly synthesized transcripts from whole transcriptomes. The kinetic rates of mRNA such as RNA synthesis and degradation in each genetically perturbed cell population were then inferred (Fig. 1a and Methods). Our method incorporates several optimizations to reduce the cell loss (Extended Data Fig. 2) and enhance the accuracy of nascent reads calling (Extended Data Fig. 3). With three levels of combinatorial indexing, *PerturbSci-Kinetics* demonstrates orders of magnitude higher throughput than previous approaches coupling metabolic labeling and single-cell RNA-seq (for example, scEU-seq, sci-fate and scNT-seq)^18–22^ (Fig. 1b).

**Extended Data Fig. 4:**
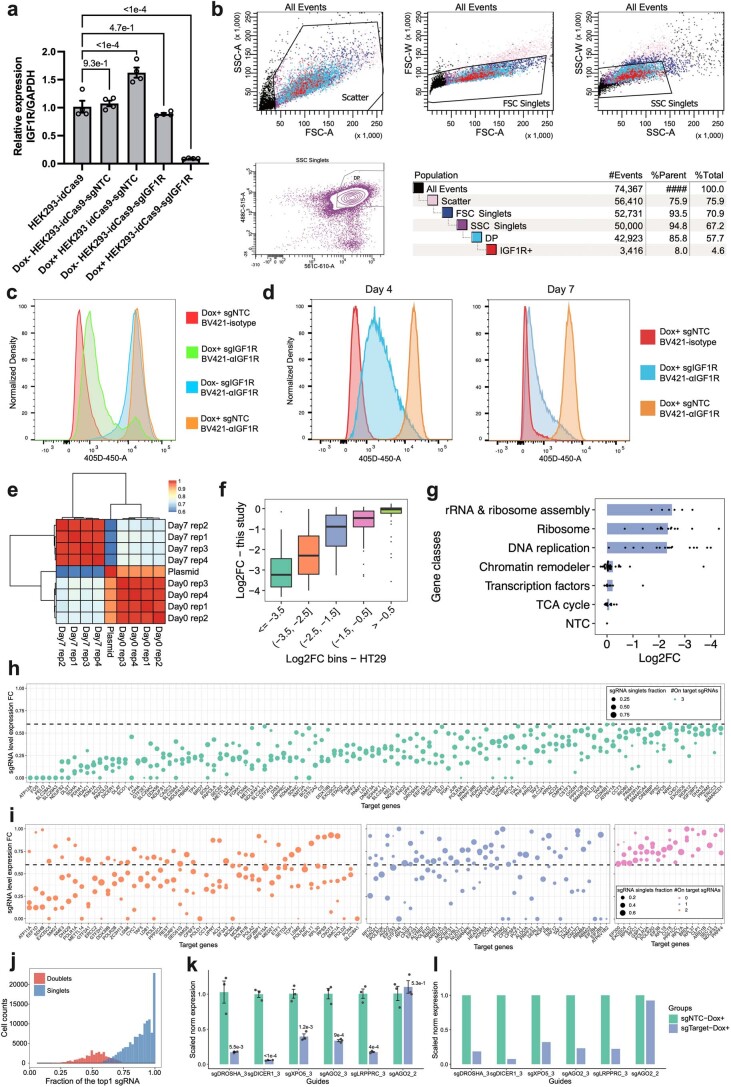
Validation of the performance of CRISPRi and quality control of bulk and single-cell *PerturbSci-Kinetics* libraries. **a–d**. Inducible *IGF1R* mRNA and protein knockdown were further validated by RT-qPCR (a) after 3-day Dox induction (n = 4 biologically independent samples, data are presented as mean ± SEM. Dunnett’s test after one-way ANOVA was performed.) and by flow cytometry (b-d). The representative gating strategy for flow cytometry is shown in (b). Cells were treated with Dox+/Dox- media for 7 days before the flow-cytometry assay (c). To find the minimal time of Dox induction with stable knockdown, sgIGF1R and sgNTC cells were induced for either 4 days or 7 days and the IGF1R abundance was examined. Isotype, isotype control. αIGFIR, anti-IGF1R. **e**. Heatmap showing the Pearson correlations of normalized sgRNA read counts between the plasmid library and bulk screen replicates. **f**. Boxplot showing the reproducible trends of deletion upon CRISPRi between the present study and a prior report^25^ (n = 10, 57, 45, 49, 68 genes in each bin from left to right). **g**. Barplot showing the knockdown of genes with higher essentiality resulted in stronger cell growth arrest. **h–i**. Dotplots showing the expression fold changes of target genes upon CRISPRi induction compared to NTC in the single-cell *PerturbSci-Kinetics* dataset. Each dot represents a sgRNA. Fold change < 0.6 was used for sgRNA filtering, and genes with 3, 2, 1, 0 on-target sgRNA(s) were visualized in b-e, respectively. FC, fold change. **j**. Histogram showing the distribution of the fraction of the most abundant sgRNA in singlets (78%) and doublet cells (22%). **k**, **l**. The accuracy of sgRNA targeting efficiency in *PerturbSci-Kinetics* was further confirmed by RT-qPCR. Individual HEK293-idCas9 clones expressing 5 sgRNAs with high efficiency and 1 off-target sgRNA were established. RT-qPCR was conducted after 3-day Dox induction (n = 3 biologically independent samples). Data are presented as mean ± SEM, and two-sided Student′s t-test were performed (k). Mean expressions of target genes in NTC and corresponding cells in the original *PerturbSci-Kinetics* dataset were exhibited (l). Boxes in boxplots indicate the median and IQR with whiskers indicating 1.5 × IQR.

As a proof of concept, we established a human HEK293 cell line with inducible dCas9-KRAB-MeCP2 (ref. ^9^) expression (HEK293-idCas9). We thoroughly validated the potent knockdown of target gene expression after doxycycline (dox) treatment (Fig. 1c and Extended Data Fig. 4a–c). Furthermore, we demonstrated the purity of the single-cell transcriptome and sgRNA capture of *PerturbSci* by profiling mixed human and mouse cells transduced with human and mouse-specific sgRNAs, respectively (Fig. 1d).

We proceeded to validate the capability of *PerturbSci-Kinetics* in capturing the three-layer readout at the single-cell level. After 4sU labeling and chemical conversion, we observed a significant enrichment of T-to-C mismatches in the mapped reads, which is consistent with findings from our previous study^20^ (Fig. 1e). A median of 22.1% of newly synthesized reads were recovered, in contrast to only 0.8% in control cells (Fig. 1f). The proportion of reads mapped to exonic regions was also significantly lower in nascent reads compared to pre-existing reads (*P* < 1 × 10^−20^, Tukey’s test after ANOVA) (Fig. 1g). Moreover, genes with a higher fraction of nascent reads were significantly enriched in highly dynamic biological processes^23^, whereas housekeeping genes were strongly enriched in genes with a lower fraction of nascent reads (Fig. 1h–i). Notably, the chemical conversion step is fully compatible with sgRNA detection. We recovered sgRNAs from 97% of chemically converted cells (a median of 62 sgRNA unique molecular identifiers (UMIs) per cell), in which 92.6% were annotated as sgRNA singlets (Fig. 1j–k).

**Fig. 2:**
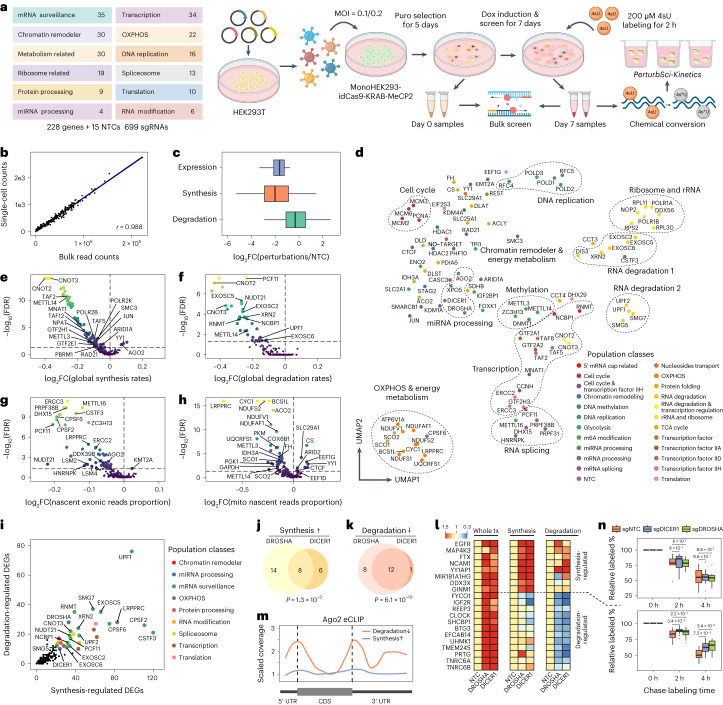
Characterizing the impact of genetic perturbations on gene-specific transcriptional and degradation dynamics with *PerturbSci-Kinetics*. **a**, Scheme of the experimental design. **b**, Scatter plot shows the correlation between perturbation-associated cell count from *PerturbSci-Kinetics* and sgRNA read counts from bulk screen libraries. **c**, Box plot showing the log_2_-transformed FCs of gene expression, synthesis rates and degradation rates of sgRNA-targeted genes (*n* = 203 genes) in perturbed cells expressing the corresponding sgRNA compared to NTC. **d**, UMAP visualization of perturbed pseudobulk whole transcriptomes profiled by *PerturbSci-Kinetics*. We aggregated single-cell transcriptomes in each perturbation, followed by dimension reduction using PCA and visualization using UMAP. Population classes: the functional categories of genes targeted in different perturbations. **e**–**h**, Scatter plots showing the extent and the significance of changes on the distributions of global synthesis (**e**), degradation (**f**), proportions of exonic reads in the nascent transcriptome (**g**) and proportions of mitochondrial nascent reads (**h**) upon perturbations compared to NTC cells. The FCs were calculated by dividing the median values of each perturbation with that of NTC cells and were log_2_ transformed. Dashed lines indicate the statistical thresholds that were used (horizontal line, −log_10_(0.05); vertical line, 0). **i**, Scatter plot showing the number of synthesis/degradation-regulated DEGs from different perturbations. nDEGs, number of DEGs. **j**,**k**, Venn diagrams showing the number of merged DEGs with significantly enhanced synthesis (**j**) or impaired degradation (**k**) between *DROSHA* and *DICER1*. One-sided Fisher’s exact tests were conducted with the alternative hypothesis that the true odds ratio is greater than 1. **l**, Heat maps showing the steady-state expression, synthesis and degradation rate changes of genes included in **j**–**k**. Tiles of each row are colored by FCs of values of perturbations relative to NTC. tx, transcriptome. **m**, Line plot showing the AGO2 binding patterns on transcripts of protein-coding genes in **j**–**k** revealed by eCLIP signal intensity. Data were obtained from a previous study^42^. Dashed lines indicate the position of the beginning of CDS (left) and the beginning of 3′ UTR (right). **n**, Box plots showing the relative proportion of labeled mRNA of transcription-regulated genes (*n* = 8) and degradation-regulated genes (*n* = 12) after chase labeling for different times in HEK293-idCas9-sgNTC, sgDROSHA and sgDICER1 cells. Two-sided Studentʼs *t*-tests were performed between knockdown groups and the NTC group. Boxes in box plots indicate the median and interquartile range (IQR), with whiskers indicating 1.5× IQR. OXPHOS, oxidative phosphorylation; Puro, puromycin.

To dissect the impact of genetic perturbations on transcriptome kinetics, we performed a *PerturbSci-Kinetics* screening on HEK293-idCas9 cells. These cells were transduced with a library of 699 sgRNAs, which included 15 no-target controls (NTCs), targeting a total of 228 genes involved in diverse biological processes (Fig. 2a and Supplementary Table 2). After a 5-d puromycin selection, we harvested a proportion of cells for bulk library preparation (referred to as ‘day 0’ samples) and induced *dCas9-KRAB-MeCP2* expression with dox for seven more days. The screening window was carefully chosen to maximize gene knockdown efficiency, minimize population dropout^8^ and allow cells to attain transcriptomic steady states^24^ (Extended Data Fig. 4d). We performed 200 µM 4sU labeling for 2 h at the end of the screening and harvested samples for both bulk and *PerturbSci-Kinetics* library preparation. As a quality control, the activation of CRISPR interference (CRISPRi) significantly altered the abundance of sgRNAs in the pool, which was consistent across replicates and aligned with previous studies^25^. For example, genes involved in essential functions (for example, DNA replication and ribosome assembly) were strongly depleted after the screening (Extended Data Fig. 4e,g). Reassuringly, the number of sgRNA singlets recovered by *PerturbSci-Kinetics* correlated well with read counts of bulk screen libraries (Pearson correlation *r* = 0.988, *P* < 2.2 × 10^−16^) (Fig. 2b).

We recovered 161,966 labeled cells with matched sgRNAs (88% of cells recovered in total), and 126,271 cells were annotated as sgRNA singlets (Extended Data Fig. 4j). Despite the shallow sequencing depth (~8,000 reads per cell), we achieved a median of 2,155 UMIs per cell. Of 699 sgRNAs, 698 were successfully recovered, with a median of 28 sgRNA UMIs per cell. Subsequently, we excluded cells containing sgRNAs that demonstrated low knockdown efficiencies (≤40% gene expression reduction compared to NTC). The RT–qPCR validation on several individual sgRNAs corroborated the accuracy of our knockdown efficiency estimates (Extended Data Fig. 4h–l). Ultimately, 98,315 cells were retained for downstream analysis, corresponding to a median of 484 cells per gene perturbation and a median knockdown efficiency of target genes at 67.7% (Fig. 2c).

**Extended Data Fig. 5:**
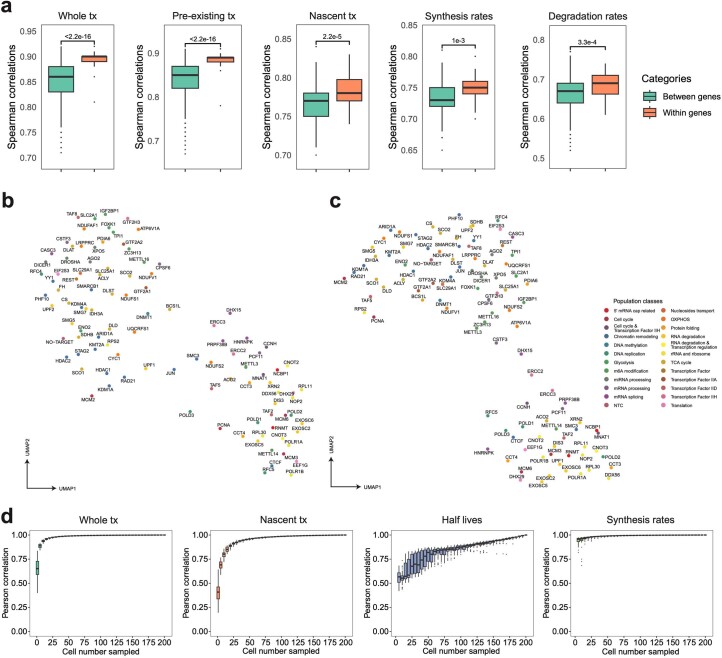
*PerturbSci-Kinetics* captures multi-layer transcriptome and RNA kinetics information upon perturbations with high fidelity. **a**. Boxplots showing the pairwise correlation coefficients of sgRNAs targeting the same/different genes, computed using aggregated whole transcriptomes, pre-existing transcriptomes, nascent transcriptomes, gene-specific synthesis rates and degradation rates. Considering the data sparsity and different cell numbers across perturbations, 150 cells per sgRNA were assembled into one pseudobulk for downstream analysis. Spearman correlation coefficients were calculated using DEGs between perturbations and NTC in the pooled screen. Two-sided Welch′s t-tests were performed. **b**, **c**. UMAP of pseudobulk perturbations by inferred synthesis rates (b) and degradation rates (c). DEGs between all perturbations-NTC pairs were combined, and their synthesis and degradation rates were calculated for each perturbation. Only genes with calculable synthesis or degradation rate in at least 75% of pseudobulk perturbations were used for dimension reduction. The top 12 and 15 principal components from the synthesis and degradation rates matrix were used for UMAP visualization, respectively. These UMAPs showed meaningful patterns. For example, RNA exosome genes (*for example, EXOSC2, EXOSC5, EXOSC6*), nonsense-mediated mRNA decay pathway members (*for example, SMG5, SMG7*), ribosomal biogenesis genes (*for example, NOP2, RPL30, RPL11, POLR1A, POLR1B*), miRNA biogenesis pathway members (*for example, DICER1, DROSHA, XPO5, and AGO2*) were in relative proximity in both UMAPs. Chromatin remodelers (*for example, HDAC1, HDAC2, STAG2, RAD21, KMT2A, KDM1A, ARID1A*) were closely clustered in synthesis rates-derived UMAP, while m6A regulators (*for example, METTL3, METTL16, ZC3H13, IGF2BP1*) and polyadenylation factors (*for example, CPSF6, CSTF3*) were closer to each other in degradation rates-derived UMAP. **d**. Boxplots showing effects of cell number on the estimation of the pseudobulk whole/nascent transcriptome expression, gene-specific half-life, and synthesis rate. We conducted 50 random samplings for each cell number on sgDROSHA cells, then we aggregated profiles of sampled cells and retrieved pseudobulk expression levels and estimated RNA dynamics rates. We calculated the Pearson correlation coefficients between each downsampled pseudobulk group and unsampled pseudobulk sample. Boxes in boxplots indicate the median and IQR with whiskers indicating 1.5× IQR.

We next quantified gene-specific synthesis and degradation rates in each perturbation using an ordinary differential equation approach^26^ (Methods). As expected, genes targeted by the CRISPRi demonstrated substantially reduced synthesis rates, whereas their degradation rates exhibited only mild alterations (Fig. 2c). As another validation, we observed significantly higher correlations of transcriptomes among sgRNAs targeting the same genes across multiple layers (for example, whole/nascent transcriptome and synthesis/degradation rates; Extended Data Fig. 5a). We then performed dimension reduction and uniform manifold approximation and projection (UMAP) visualization^27^ on aggregated whole transcriptomes of each perturbation. Perturbations targeting paralogous genes (for example, *EXOSC5* and *EXOSC6*) or related biological processes (for example, RNA degradation and energy metabolism) were readily clustered together (Fig. 2d). Similar analyses on gene-specific synthesis/degradation rates managed to group perturbations by their functions (Extended Data Fig. 5b,c). Furthermore, by aggregating profiles of single cells carrying sgRNAs that target the same gene, we achieved robust estimations for both whole/nascent transcriptomes as well as transcriptome kinetic rates (Extended Data Fig. 5d).

**Extended Data Fig. 6:**
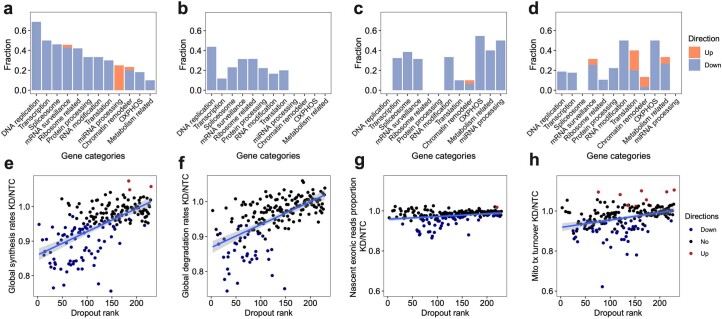
A systematic view of the effects of perturbations on global synthesis rates, global degradation rates, exonic reads ratio, and mitochondrial turnover rates. **a–d**. For each gene category, we calculated the fraction of genetic perturbations associated with significant changes in global synthesis rates (a), global degradation rates (b), proportions of exonic reads in the nascent transcriptome (c), and proportions of mitochondrial nascent reads (d). Overall global transcription could be affected by more genes than degradation. Perturbation on essential genes, such as DNA replication genes, could affect both global synthesis and degradation. Perturbations on chromatin remodelers only specifically impaired the global synthesis rates but not the degradation rates, supporting the established theory that gene expression is regulated by chromatin folding. In addition to the enrichment of genes in transcription, spliceosome and mRNA surveillance, perturbation on OXPHOS genes and metabolism-related genes also affected the RNA processing, consistent with the fact that 5′ capping, 3′ polyadenylation, and RNA splicing are highly energy-dependent processes. That knockdown of OXPHOS genes and metabolism-related genes could reduce the mitochondrial transcriptome dynamics and also supported the complex feedback mechanisms between energy metabolism and mitochondrial transcription^63^. **e–h**. Scatterplots showing the relationships between dropout effects and global synthesis rates (e), global degradation rates (f), proportions of exonic reads in the nascent transcriptome (g), and mitochondrial RNA turnover (h). A linear regression line was fitted and ±95% confidence intervals are visualized for each metric. Dropout rank, the ascending rank of gene-level sgRNA counts log2FC from the bulk screen. Directions were assigned as shown in Fig. 2e–h. Both global synthesis and degradation rates showed strong negative correlations with dropout, indicating knocking out essential genes generally resulted in impaired global RNA synthesis and degradation. In contrast, proportions of exonic reads in the nascent transcriptome were much more stable across perturbations, and were only specifically affected by genes functioning in RNA processing. Proportions of mitochondrial nascent reads were also prone to be affected by genetic perturbation, but directions of changes depend more on the functions of perturbed genes than the essentiality of genes.

We then investigated how genetic perturbations influence global transcriptome dynamics (Fig. 2e–g, Extended Data Fig. 6a–c,e–g and Supplementary Tables 5–7). As expected, the knockdown of genes encoding proteins involved in transcription initiation (for example, *GTF2E1* and *TAF2*), mRNA synthesis (for example, *POLR2B* and *POLR2K*) and chromatin remodeling (for example, *SMC3* and *RAD21*) significantly downregulated the global synthesis rates but not the degradation rates. Conversely, perturbations targeting critical biological processes, such as DNA replication (for example, *POLA2* and *POLD1*), ribosome synthesis and rRNA processing (for example, *POLR1A*, *POLR1B*, *RPL11* and *RPS15A*) and mRNA and protein processing (for example, *CNOT2*, *CNOT3*, *CCT3* and *CCT4*), reduced both global RNA synthesis and degradation, indicating a compensatory mechanism for maintaining transcriptome homeostasis^28^ (Fig. 2e,f). Moreover, we noted significant reductions in exonic read fractions in nascent transcriptomes after perturbations related to RNA processing (for example, *NCBP1*, *LSM2*, *LSM4*, *CPSF2* and *CPSF6*) and energy metabolism (for example, *GAPDH* and *NDUFS2*), signifying dysregulated splicing dynamics (Fig. 2g).

**Extended Data Fig. 7:**
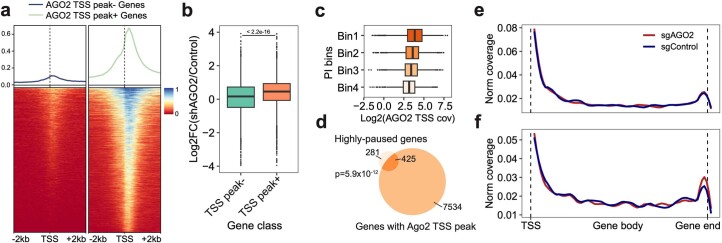
AGO2 functions as a transcriptional repressor by arresting transcription at the pausing status. **a**. The density plot (top) and heatmap (bottom) show the density of AGO2 ChIP-seq reads around TSS of genes with or without enriched AGO2 TSS binding peaks. **b**. Boxplot showing the log2FC of gene expression between *AGO2*-silenced and control groups of genes with (n = 7315 genes) or without AGO2 TSS binding peaks (n = 3615 genes). Two-sided Wilcoxon rank sum test was performed. **c**. Boxplot displaying the positive correlation between PI of genes and normalized AGO2 ChIP-seq coverage within corresponding TSS regions. **c**, **d**. Genes were separated into 4 bins based on the average ranks of PI in two replicates (Methods). The Venn diagram highlights the significant association between *AGO2* TSS binding and the strong pausing status of genes. One-sided Fisher′s Exact Test was conducted with the alternative hypothesis that the true odds ratio is greater than 1. Highly-paused genes, genes with top 10% of average PI ranks. **e**, **f**. Highly-paused genes were split into two groups, 1) significantly-upregulated genes upon *AGO2* knockdown or 2) genes without significant expression changes. We then calculated the nascent RNA coverage of these two groups of genes in sgNTC and sgAGO2 cells. Notably, only genes in group 1 displayed increased 3′ end enrichment upon *AGO2* knockdown (f). Boxes in boxplots indicate the median and IQR with whiskers indicating 1.5× IQR.

Interestingly, the knockdown of *AGO2*, a recognized post-transcriptional regulator^29^, led to an increase in global synthesis, suggesting its potential role in transcriptional repression (Fig. 2e). The re-analysis of public datasets^30,31^ corroborated our observation. Specifically, genes exhibiting enriched AGO2 binding at transcription start sites (TSSs) were markedly upregulated after *AGO2* silencing (Extended Data Fig. 7a,b). Additionally, the enrichment of AGO2 binding was observed immediately downstream of the TSS and was positively correlated with transcriptional pausing (Extended Data Fig. 7a–d). For validation, we employed SLAM-seq^32^ to examine the transcriptomic response after *AGO2* knockdown, identifying 78 highly paused genes significantly upregulated (FDR of 0.05). Notably, the nascent RNA of these genes showed increased 3′ end coverages compared to NTC, indicative of more efficient transcriptional elongation (Extended Data Fig. 7e,f). Collectively, our integrated analyses support the unconventional function of AGO2 in transcriptional repression.

**Extended Data Fig. 8:**
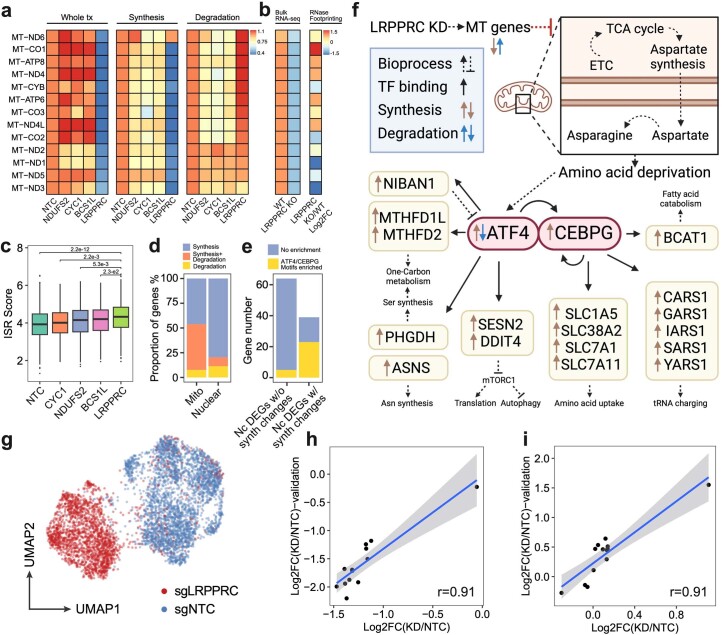
*PerturbSci-kinetics* identified LRPPRC as the master regulator of mitochondrial RNA dynamics. **a**. Heatmap showing the relative FC of gene expression, synthesis and degradation rates of mitochondrial protein-coding genes upon *NDUFS2*, *CYC1*, *BCS1L* and *LRPPRC* knockdown compared to NTC cells. **b**. The heatmap (left) showed mean z-scored mitochondrial gene expression changes between wild-type and *LRPPRC*-knockout mice heart tissue samples reported by Siira, S.J., et al.^33^. The DEG statistical examination was conducted by the original study. The heatmap (right) showed the FC of the mRNA secondary structure increase upon *LRPPRC* knockdown observed in the same prior report^33^, which positively correlated with the accelerated degradation of mitochondrial genes detected in our study (coefficient of Pearson correlation = 0.708, p-value = 6.8e-3). **c**. Boxplot showing the distribution of integrated stress response scores of single cells (n = 2758, 478, 768, 631, 504 cells in each group from left to right). Dunnett’s test after one-way ANOVA was performed. ISR, integrated stress response. ISR score, the average normalized expression of genes within the ISR transcription program identified by Genome-wide Perturb-seq^8^. **d**. Barplot showing the fraction of genes regulated by synthesis, degradation or both in mitochondrial/nuclear-encoded DEGs. **e**. Barplot showing the enrichment of ATF4/CEBPG motifs at promoter regions of DEGs with/without significant synthesis changes. We identified two transcription factors (*ATF4* and *CEBPG*) that were significantly upregulated upon *LRPPRC* knockdown, and motifs of their protein product were significantly over-represented in TSS regions of the synthesis-regulated nuclear-encoded DEGs. Nc DEGs w/o synth changes, Nuclear-encoded DEGs without synthesis changes. Nc DEGs w/ synth changes, Nuclear-encoded DEGs with synthesis changes. **f**. The transcriptional regulatory network in *LRPPRC* perturbation inferred from our analysis. It was consistent with the prior study^64^ that *ATF4* was regulated at both transcriptional and post-transcriptional levels. **g**. Single-cell UMAP of HEK293-idCas9-sgNTC/sgLRPPRC cells in the validation dataset. **h**, **i**. Correlations of synthesis and degradation rate changes of mitochondrial mRNA upon *LRPPRC* knockdown between the original screen and the validation dataset. A linear regression line was fitted and ±95% confidence intervals are visualized for each metric. r, coefficient of Pearson correlation. Boxes in boxplots indicate the median and IQR with whiskers indicating 1.5× IQR.

We next investigated regulators of mitochondrial RNA dynamics by quantifying the fraction of nascent reads in single-cell mitochondrial transcriptomes. A significant reduction in mitochondrial transcriptome turnover was observed after perturbing metabolism-associated genes, including those encoding proteins involved in glycolysis (for example, *GAPDH*, *FH* and *PKM*), the tricarboxylic acid (TCA) cycle (for example, *ACO2* and *IDH3A*) and oxidative phosphorylation (for example, *NDUFS2* and *COX6B1*) (Fig. 2h, Extended Data Fig. 6d,h and Supplementary Table 8). Notably, LRPPRC emerged as a key mitochondrial RNA dynamics regulator, as its knockdown led to substantial reduction in both turnover rates and expression levels across most mitochondrial protein-coding genes and mitochondrial functional defects (Extended Data Fig. 8a–c and Supplementary Table 9). In contrast, nuclear-encoded genes were primarily regulated at the transcriptional level upon *LRPPRC* knockdown (Extended Data Fig. 8d–f). These kinetic changes in mitochondrial mRNA were validated through an independent *PerturbSci-Kinetic* experiment that profiled with *LRPPRC* knockdown (Extended Data Fig. 8g–i). Recent studies reported similar findings, observing impaired mitochondrial gene expression and mitochondrial functional defects in the hearts of *LRPPRC* knockout mice^33^ and in brown adipocyte-specific *LRPPRC* knockout mice^34^. This further corroborates the essential role of LRPPRC in maintaining mitochondrial mRNA homeostasis.

**Extended Data Fig. 9:**
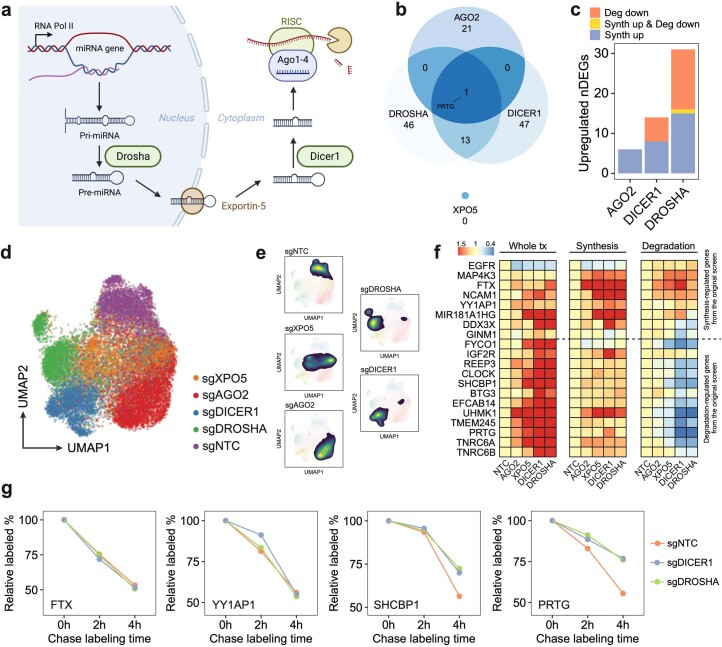
*PerturbSci-Kinetics* identified post-transcriptional gene expression regulations by perturbing miRNA biogenesis pathway. **a**. Illustration of the canonical miRNA biogenesis pathway. After the transcription of miRNA host genes, the primary miRNA (pri-miRNA) forms into a hairpin and is processed by DROSHA. Processed precursor miRNA (pre-miRNA) is transported to the cytoplasm by Exportin-5. The stem loop is cleaved by DICER1, and one strand of the double-stranded short RNA is selected and loaded into the RISC for targeting mRNA^35^. **b**. Venn diagram showing the overlap of upregulated DEGs across perturbations on four genes encoding main members of the miRNA pathway. The knockdown of *DROSHA* and *DICER1* in this pathway resulted in significantly overlapped DEGs (p-value = 2.2e-16, one-sided Fisher’s exact test). In contrast, *AGO2* knockdown resulted in more unique transcriptome features. *XPO5* knockdown showed no upregulated DEGs, consistent with a previous report in which *XPO5* silencing minimally perturbed the miRNA biogenesis^36^. **c**. Bar plot showing the fraction of upregulated DEGs driven by synthesis/degradation changes upon *DROSHA*, *DICER1*, and *AGO2* perturbations. While *DROSHA* and *DICER1* knockdown resulted in increased synthesis and reduced degradation, *AGO2* knockdown only affected gene expression transcriptionally, consistent with our finding that *AGO2* knockdown resulted in a global increase of synthesis rates (Fig. 2e), and further supported its roles in nuclear transcription regulation^65–67^. As DROSHA is upstream of DICER1 in the pathway, we observed stronger effects of *DROSHA* knockdown than *DICER1* knockdown, which was supported by the previous study^36^. **d**, **e**. UMAP of sgNTC cells and single cells with individual miRNA biogenesis pathway genes knockdown. **f**. Reproducible steady-state expression, synthesis rate, and degradation rate changes of synthesis/degradation-regulated genes in the validation dataset. **g**. Example genes showing unchanged (transcription-regulated genes: *FTX, YY1AP1*) and enhanced (degradation-regulated genes: *SHCBP1, PRTG*) mRNA stability upon *DROSHA*/*DICER* knockdown. After long term 4sU labeling on Dox-induced HEK293-idCas9-sgNTC, sgDROSHA, sgDICER cells, uridine chase was performed. 3′end SLAM-seq was performed to directly track the degradation of labeled mRNA. The fraction of labeled read counts of individual genes at each time point were normalized by their labeled fractions at 0h.

To further demonstrate the unique capacity of *PerturbSci-Kinetics* in unraveling the regulatory mechanisms that govern gene expression control, we identified 14,618 differentially expressed genes (DEGs) across perturbations, with 22.9% of them exhibiting significant changes in their synthesis or degradation rates (Supplementary Tables 10 and 11 and Methods). Among these, DEGs regulated by RNA degradation were associated with perturbations in mRNA surveillance/processing genes (Fig. 2i). For instance, our study revealed a set of significantly overlapped DEGs upon knockdown of *DROSHA* and *DICER1* (refs. ^35,36^), genes encoding two crucial RNases in the miRNA biogenesis pathway^37^ (Extended Data Fig. 9a–c). These DEGs were regulated through distinct mechanisms: some genes were regulated by decreased degradation (for example, genes encoding miRNA-mediated silencing complex (RISC) components: *TNRC6A* and *TNRC6B*), whereas others are regulated through increased transcription (for example, miRNA host genes: *MIR181A1HG* and *FTX*; genes encoding protein involved in miRNA biogenesis: *DDX3X*) (Fig. 2j–l and Supplementary Table 12). The RNA-binding pattern of AGO2, a core component of RISC for miRNA-mediated mRNA degradation^38^, further validated our findings, exhibiting a strong enrichment in the untranslated regions (UTRs) of transcripts from degradation-regulated genes but not in synthesis-regulated genes (Fig. 2m). This finding was further substantiated through *PerturbSci-Kinetics* profiling on individual sgRNA knockdown clones and SLAM-seq after 4sU chase labeling^32^ (Fig. 2n and Extended Data Fig. 9d–g).

**Extended Data Fig. 10:**
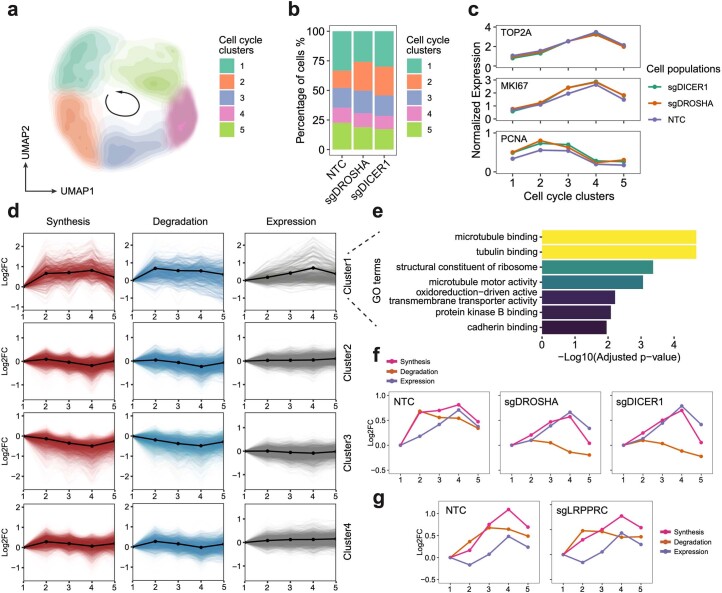
*PerturbSci-Kinetics* enables dissecting the effects of perturbations on cell cycle-dependent RNA dynamics. **a**. UMAP embedding of cells with miRNA pathway genes knockdowns and NTC cells reflected the cell-cycle progression. **b**. Stacked barplot showing the cell cycle distribution of cells from each perturbation. **c**. The expression changes of cell cycle marker genes in cell cycle clusters. **d**. The cell cycle time-course synthesis rates, degradation rates, and steady-state expression changes of 4 gene clusters. Solid lines with dots, the mean values and the average trend of all genes within the cluster. **e**. The top enriched GO terms of genes in the cluster 1 identified in GO enrichment analysis. **f**. Averaged trends of cell cycle time-course synthesis rates, degradation rates, and steady-state expression changes of cluster 1 genes in HEK293-idCas9-sgNTC, sgDROSHA, sgDICER1 cells. **g**. Averaged trends of cell cycle time-course synthesis rates, degradation rates, and steady-state expression changes of genes in cluster 1 in HEK293-idCas9-sgNTC and sgLRPPRC cells. Considering potential strong batch effects from distinct genetic perturbation, cell cycle clustering analysis in (g) was performed independently of (a), and cell cycle clusters in (g) were not fully synchronized with clusters in (f).

Finally, we delved into the effects of genetic perturbations on RNA dynamics during cell cycle progression. Using our validation dataset, we separated cells into five clusters representing different cell cycle stages using cell-cycle-related genes^39^ (Extended Data Fig. 10a–c), and we then calculated stage-specific kinetic rates of genes. Employing mfuzz clustering^40^, we identified four gene clusters displaying discrepant cell cycle timecourse synthesis dynamics patterns. Among these, only genes in cluster 1 exhibited evident steady-state expression fluctuations (Extended Data Fig. 10d). Although their synthesis and degradation rates both increased in early cell cycle phase, the synthesis rates outpaced the degradation rates, leading to an increase in steady-state mRNA levels from the S to the G2M stage. Gene Ontology (GO) term analysis further supported the crucial roles of proteins encoded by these genes in cell cycle (Extended Data Fig. 10e). Interestingly, in cells with *DROSHA* and *DICER1* knockdown, we observed a similar steady-state expression pattern for genes in cluster 1 but with unresponsive degradation and compensated synthesis during cell cycle progression (Extended Data Fig. 10f), suggesting the existence of synthesis/degradation feedback loops for gene regulation. In contrast, *LRPPRC* knockdown did not impact cell-cycle-dependent RNA degradation dynamics (Extended Data Fig. 10g), aligning with our results that it specifically affects mitochondrial mRNA stability. Together, our study emphasizes the coordinated regulation of gene expression throughout the cell cycle progression and highlights the presence of intricate feedback loops between RNA synthesis and degradation.

In summary, *PerturbSci-Kinetics* allows for the quantitative analysis of the genome-wide mRNA kinetics across genetic perturbations in a massively parallel manner. Of note, there are several potential limitations to consider. First, extended 4sU labeling might impact cell states and potentially hinder the identification of sgRNA sequences. To mitigate this, we opted for a relatively short-term (2 h) treatment to minimize such effects. Second, RNA dynamics identified by *PerturbSci-Kinetics* may not directly reflect causality in gene regulation, partly due to the gradual nature of CRISPRi-based gene knockdown. This limitation could be mitigated by coupling the technique with large-scale chemical perturbations. Third, the perturbation of essential genes might lead to significant dropout, affecting dynamic rate estimations due to limited cells and reads. Moreover, apoptosis-triggered mRNA decay might further complicate the analysis^41^. Therefore, we recommend excluding genetic perturbations that lead to either strong dropout effects or substantial disruption of cell cycle distribution during RNA dynamics analysis.

Despite these limitations, our findings illuminate the distinct advantages of *PerturbSci-Kinetics* over conventional assays. Its multi-layer readout provides a comprehensive perspective on gene expression and RNA dynamics in response to genetic perturbations, facilitating high-throughput and parallel characterization of elements that govern gene-specific RNA dynamics. Moreover, given the low cost and high sensitivity of *PerturbSci*, we envision the potential to systematically dissect cell-type-specific gene regulatory networks across various biological contexts with an unparalleled scale and resolution.

## Methods

### Cell culture

The 3T3-L1-CRISPRi cell line was obtained from the Tissue Culture facility at the University of California, Berkeley. The HEK293 cell line was a gift from the Scott Keeney laboratory at Memorial Sloan Kettering Cancer Center. The HEK293T cell line and the NIH/3T3 cell line were obtained from the American Type Culture Collection. All cells were maintained at 37 °C and 5% CO_2_ in high glucose DMEM medium supplemented with l-glutamine and sodium pyruvate (Gibco, 11995065) and 10% FBS (Sigma-Aldrich, F4135).

### Cell lines generation

To generate HEK293 cells with dox-inducible dCas9-KRAB-MeCP2 expression, the lentiviral plasmid Lenti-idCas9-KRAB-MeCP2-T2A-mCherry-Neo was constructed. After sequencing validation, the lentivirus was produced by co-transfecting Lenti-idCas9-KRAB-MeCP2-T2A-mCherry-Neo with psPAX2 (Addgene, 12260) and pMD2.G (Addgene, 12259) into low-passage HEK293T cells in a 10-cm dish using Polyjet (SignaGen, SL100688). After lentiviral titration, HEK293 cells were transduced at a multiplicity of infection (MOI) of 0.2 for 48 h. Cells were treated with 1 µg ml^−1^ dox (Sigma-Aldrich, D5207) for 48 h, and single cells with strong mCherry fluorescence were sorted for monoclonal generation.

The polyclone 3T3-CRISPRi cell line was generated in a similar way. pHR-SFFV-dCas9-BFP-KRAB (Addgene, 46911) was co-transfected with psPAX2 and pMD2.G to generate dCas9-expressing lentivirus, and the transduction at MOI = 0.2 was performed on 3T3 cells. BFP^hi^ cells (top 35% in the BFP^+^ population) were sorted, and the sorting was repeated twice more after cell expansion to enrich cells with strong dCas9 expression.

### Single-gene knockdown and efficacy examination

CROP-seq-opti-Puro-T2A-GFP was assembled by adding a T2A-GFP downstream of puromycin-resistant protein coding sequence on the CROP-seq-opti plasmid (Addgene, 106280). Oligos for individual guides cloning were ordered from Integrated DNA Technologies (IDT) with the following design:

Plus strand: 5′-CACCG[20 bp sgRNA plus strand sequence]-3′

Minus strand: 5′-AAAC[20 bp sgRNA minus strand sequence]C-3′

Oligos were phosphorylated using T4 PNK (New England Biolabs (NEB), M0201S) and were annealed. The CROP-seq-opti-Puro-T2A-GFP was digested by Esp3I (NEB, R0734L), and then the linearized backbone and the annealed duplex were ligated using the Blunt/TA Ligase Master Mix (NEB, M0367S). Transformation, clone amplification, sequencing validation, lentivirus generation and titer measurement were done as stated above.

Mouse 3T3-L1-CRISPRi cells and 3T3-CRISPRi cells were transduced with the lentivirus expressing NTC sgRNA or sgRNA targeting *Fto*. Human HEK293-idCas9 cells were transduced with lentivirus expressing NTC sgRNA or sgRNA targeting *IGF1R* during technique development, and HEK293-idCas9-sgXPO5, sgAGO2, sgDROSHA, sgDICER1 and sgLRPPRC cell lines were later established for validating significant hits from the screen. Transduction was carried out at MOI = 0.2 with 8 µg ml^−1^ of polybrene for 48 h. Transduced cells were then selected by either fluorescence-activated cell sorting (FACS) or puromycin treatment.

For RT–qPCR validation, primer pairs were selected from PrimerBank (https://pga.mgh.harvard.edu/primerbank/) and were synthesized by IDT. Total RNA of each sample was extracted using the RNeasy Mini Kit (Qiagen, 74104). Then, 1 µg of total RNA was reverse transcribed, and PowerUp SYBR Green Master Mix (Thermo Fisher Scientific, A25742) was used for RT–qPCR following the manufacturer’s instructions. The data were analyzed and visualized by GraphPad Prism (9.2.0) software.

For flow cytometry validation, 1 × 10^6^ cells of each sample were harvested and resuspended in 100 µl of PBS/0.1% sodium azide/2% FBS. BV421 Mouse Anti-Human CD221 (BD Biosciences, 565966) and BV421 Mouse IgG1 κ Isotype Control (BD Biosciences, 562438) at the final concentration of 10 µg ml^−1^ were added, and reactions were incubated at 4 °C in the dark with rotation for 30 min. Cells were then washed twice using PBS/0.1% sodium azide/2% FBS, and fluorescence signals were recorded. The data were analyzed and visualized by FlowJo (10.8.1) software.

### Construction of the pooled sgRNA library

Genes to be included in our sgRNA library were selected based on the following considerations. (1) Essential and non-essential genes were identified using the bulk CRISPR screen data from a previous report^25^ and Depmap^43^, and both were included in the gene set. (2) To validate the ability of *PerturbSci-kinetics* to characterize gene-specific RNA dynamics, we selected genes involved in transcription, chromatin remodeling, RNA processing and mRNA decay based on GO terms^44^ and KEGG pathways^45^. (3) We ensured that all selected genes were expressed in the cell line to be used in our study. An in-house HEK293 EasySci-RNA dataset was used to select expressing genes that met criteria 1 and 2.

sgRNA sequences targeting genes of interest were obtained from an established optimized CRISPRi sgRNA library (set A)^25^. Finally, 684 sgRNAs targeting 228 genes (three sgRNAs per gene) and 15 NTCs were included in the present study.

The single-stranded sgRNA library was synthesized in a pooled manner by IDT in the following format:

5′-GGCTTTATATATCTTGTGGAAAGGACGAAACACCG[20 bp sgRNA plus strand sequence]GTTTAAGAGCTATGCTGGAAACAGCATAGCAAGTT-3′

Next, 100 ng of oligo pool was amplified by PCR using primers targeting the 5′ homology arm (HA) and the 3′ HA. The PCR product was purified, and the insert was cloned into Esp3I-digested CROP-seq-opti-Puro-T2A-GFP by Gibson Assembly. In parallel, a control Gibson Assembly reaction containing only the backbone was set. Both reactions were cleaned up by 0.75× AMPure beads (Beckman Coulter, A63882) and eluted in 5 µl of EB buffer (Qiagen, 19086) and then were transformed into Endura electrocompetent cells (Lucigen, 602422) by electroporation (Gene Pulser Xcell Electroporation System; Bio-Rad, 1652662). After recovery, cells of each reaction were spread onto a 245-mm square agarose plate (Corning, 431111) with 100 µg ml^−1^ carbenicillin (Thermo Fisher Scientific, 10177012) and were then grown at 32 °C for 13 h. All colonies from each reaction were scraped from the plates, and the CROP-seq-opti-Puro-T2A-GFP-sgRNA plasmid library was extracted using ZymoPURE II Plasmid Midiprep Kit (Zymo Research, D4200). The lentiviral library was generated as stated.

### The pooled *PerturbSci-Kinetics* screen experiment

For each replicate, 7 × 10^6^ uninduced HEK293-idCas9 cells were seeded. Two replicates were transduced at MOI = 0.1, and another two replicates were transduced at MOI = 0.2. At least 1,000× coverage was kept throughout the cell culture. At the end of the puromycin selection, we harvested 1.4 × 10^6^ cells in each replicate (2,000× coverage per sgRNA) as day 0 samples of the bulk screen and pellet down at 500*g* and 4 °C for 5 min for genomic DNA extraction. For the rest of the cells, the dCas9-KRAB-MeCP2 expression was induced by adding dox at the final concentration of 1 µg ml^−1^, and l-glutamine^+^, sodium pyruvate^−^, high glucose DMEM was used to sensitize cells to perturbations on energy metabolism genes. Cells were cultured for an additional 7 d. On day 7, 6 ml of the original media from each plate was mixed with 6 µl of 200 mM 4sU (Sigma-Aldrich, T4509-25MG) dissolved in DMSO (VWR, 97063-136) and was put back for nascent RNA metabolic labeling. After 2 h of treatment, 1.4 × 10^6^ cells in each replicate were harvested as day 7 samples of the bulk screen, and the rest of the cells were fixed for *PerturbSci-Kinetics* profiling (see the next subsection).

Genomic DNA of bulk screen samples was extracted using Quick-DNA Miniprep Plus Kit (Zymo Research, D4068T) following the manufacturer’s instructions. The bulk screen libraries were amplified from genomic DNA extracted using custom primers (Supplementary Note 2) for sequencing.

Step-by-step protocols for *PerturbSci-Kinetics* library preparation are included in Supplementary Note 1.

### 4sU pulse and chase labeling and SLAM-seq

HEK293-idCas9-sgAGO2 and sgNTC cells were induced with dox for 7 d in 10-cm dishes, and cells were labeled with 600 µM 4sU for 20 min before total RNA extraction. HEK293-idCas9-sgDROSHA, sgDICER1 and sgNTC cells were induced with dox for 7 d and were treated with dox^+^ medium containing 100 µM 4sU for 18 h. The medium was refreshed every 6 h. Then, chase labeling was performed by using medium with 10 mM uridine (Sigma-Aldrich, U3750-1G). After 2-h and 4-h incubation, total RNA was extracted.

Next, 2–5 µg of total RNA from each sample was used for chemical conversion. RNA was diluted into 15 µl and mixed with 5 µl of 100 mM iodoacetamide (IAA), 5 µl of NaPO_4_ (pH 8.0, 500 mM) buffer and 25 µl of DMSO. The reaction was incubated at 50 °C for 15 min and was then quenched with 1 µl of 1 M DTT. After RNA purification using the Monarch RNA Cleanup Kit (NEB, T2030L), samples were immediately used for library construction.

Full-length and 3′ end bulk SLAM-seq was used for different experimental purposes. For full-length bulk SLAM-seq library construction, the CRISPRclean Stranded Total RNA Prep with rRNA Depletion Kit (Jumpcode Genomics, KIT1014) was used. For 3′ end bulk SLAM-seq library construction, an in-house 3′ end library preparation workflow was used. In brief, 250–500 ng of total mRNA was mixed with 1 µl of 100 µM oligodT primer (ACGACGCTCTTCCGATCTNNNNNNNNNNTTTTTTTTTTTTTTT), 1 µl of 10 mM each dNTP mix and 0.5 µl of SUPERase In, and the volume was adjusted to 15 µl with water. After RNA priming at 55 °C for 5 min, 4 µl of 5×RT buffer and 1 µl of Maxima H Minus Reverse Transcriptase (Thermo Fisher Scientific, EP0753) were added to the reaction, and reverse transcription was performed as recommended by the manufacturer. After 0.6× AMPure beads purification, second strand synthesis (NEB, E6111L) was carried out by 1-h incubation at 16 °C, and then cDNA was purified by 0.6× AMPure beads. After Read2 tagmentation on 10 ng of cDNA using 1:20 v/v Nextera Read2-Tn5, the reaction was quenched, and the final library was prepared as EasySci-RNA^10^.

### Reads processing

For bulk CRISPR screen libraries, BCL files were demultiplexed into FASTQ files based on index 7 barcodes. Reads for each sample were further extracted by index 5 barcode matching. Every read pair was matched against two constant sequences (Read1: 11–25 bp; Read2: 11–25 bp) to remove artifacts. For all matching steps, a maximum of one mismatch was allowed. Finally, sgRNA sequences were extracted from filtered read pairs (at 26–45 bp of Read1) and assigned to sgRNA identities with no mismatch allowed, and read counts matrices at sgRNA and gene levels were quantified using Python (2.7).

For *PerturbSci-Kinetics*, after demultiplexing on index 7, Read1 was matched against a constant sequence on the sgRNA capture primer to remove unspecific priming, and cell barcodes and UMI sequences sequenced in Read1 were added to the headers of the FASTQ files of Read2, which were retained for further processing. After trimming poly(A) sequences and low-quality bases from Read2 by Trim_Galore (0.6.7)^46^, reads were aligned to a customized reference genome consisting of a complete hg38 reference genome (GRCh38.p13 from GENCODE) and the *dCas9-KRAB-MeCP2* sequence using STAR (2.7.9a)^47^. Reads with mapping score ≥30 were selected by SAMtools (1.13)^48^. Then, de-duplication at the single-cell level was performed based on the UMI sequences and the alignment location, and retained reads were split into SAM files per cell. These single-cell SAM files were converted into alignment TSV files using the sam2tsv function in jvarkit (d29b24f)^49^. After background single-nucleotide polymorphism (SNP) removal, we considered T > C mismatches with the CIGAR string ‘M’ and quality scores >45 as 4sU site. Only reads with >30% of T > C mutations among all mismatches were identified as nascent reads, and the list of reads was extracted from single-cell whole-transcriptome SAM files by Picard (2.27.4)^50^. Finally, single-cell whole/nascent transcriptome gene × cell count matrices were constructed by assigning reads to genes^51^.

Read1 and Read2 of *PerturbSci-Kinetics* sgRNA libraries were matched against constant sequences, respectively, allowing a maximum of one mismatch. For each filtered read pair, cell barcode, sgRNA sequence and UMI were extracted from designed positions. Extracted sgRNA sequences with a maximum of one mismatch from the sgRNA library were accepted and corrected, and the corresponding UMI was used for de-duplication. De-duplication was performed by collapsing identical UMI sequences of each individual corrected sgRNA under a unique cell barcode. Cells with overall sgRNA UMI counts higher than 10 were maintained, and the sgRNA × cell count matrix was constructed.

SLAM-seq reads were processed similarly. In brief, for 3′ end SLAM-seq, UMI sequences in Read1 were extracted and attached to the headers of Read2 by UMI-tools (1.1.2)^52^, and only Read2 was further processed. After poly(A) and low-quality base trimming by Trim_Galore, reads were aligned to the hg38 reference genome by STAR. In the scenario of high-concentration 4sU labeling, more loose alignment parameters were used (–outFilterMatchNminOverLread 0.2–outFilterScoreMinOverLread 0.2). Reads were filtered by SAMtools, and PCR duplicates in passed reads were further removed by UMI-tools. Nascent reads were identified and extracted, and gene counting on both whole transcriptome and nascent transcriptome were performed as mentioned above but at the sample level. For full-length SLAM-seq, reads were processed similarly, but paired-end reads were retained.

### sgRNA singlets identification and off-target sgRNA removal

Cells with at least 300 whole-transcriptome UMIs, 200 genes, 10 sgRNA UMIs and unannotated reads ratio <40% were kept. sgRNA singlets were assigned based on the following criteria: the most abundant sgRNA in the cell took ≥60% of total sgRNA counts and was at least three-fold of the second most abundant sgRNA.

Target genes with the number of cells perturbed ≥50 were kept. The knockdown efficiency was calculated at the individual sgRNA level to remove potential off-target or inefficient sgRNAs: whole transcriptomes of cells receiving the same sgRNA were merged and normalized by counts per million (CPM), and then the fold changes (FCs) of the target gene expressions were calculated by comparing the normalized expression levels between corresponding perturbations and NTC. sgRNAs with ≥40% of target gene expression reduction relative to NTC were regarded as ‘effective sgRNAs’, and singlets receiving these sgRNAs were kept as ‘on-target cells’. Downstream analyses were done at the target gene level by analyzing all cells receiving different sgRNAs targeting the same gene.

### UMAP embedding on pseudo-cells

The count matrix of the ‘on-target’ cells described above was loaded into Seurat^27^, and DEGs of each perturbation (compared to NTC) were retrieved. Cells from perturbations with ≥1 DEGs and cells from genetic perturbations involved in similar pathways of the top perturbations were kept. The FCs of the normalized gene expression between perturbations and NTC were calculated and were binned based on the gene-specific expression levels in NTC. The top 3% of genes showing the highest FCs within each bin were selected and merged as features for principal component analysis (PCA). The top nine principal components (PCs) were used as input for UMAP embedding.

### Differential expression analysis

Pairwise differential expression analyses between each perturbation and NTC cells were performed by Monocle 2 (ref. ^53^). We selected significant hits (false discovery rate (FDR) < 0.05) with a ≥1.5-fold expression difference and CPM ≥5 in at least one of the tested cell pairs. More stringent criteria were used to obtain DEGs with high confidence: significant hits (FDR < 0.05) with a ≥1.5-fold expression difference and CPM ≥50 in at least one of the tested cell pairs were kept. For bulk RNA-seq libraries, genes with a minimum of ten raw counts in at least one sample and expressed in at least half of samples were kept, and EdgeR^54^ was used for bulk RNA-seq DEG analysis. Significant hits were selected at an FDR < 0.05 level.

### Synthesis and degradation rates calculation

After the induction of CRISPRi for 7 d, we assumed that new transcriptomic steady states had been established at the perturbation level before the 4sU labeling, and the labeling did not disturb these new transcriptomic steady states. The following RNA dynamics differential equation was used for synthesis and degradation rates calculation, similarly to the previous study^26^:

1 $$\frac{d(R)}{d(t)}=\alpha -R\times \beta$$

in which *R* is the mRNA abundance of each gene; *α* is the synthesis rate of this gene; and *β* is the degradation rate of this gene. Because the RNA synthesis follows the zero-order kinetics, and RNA degradation follows the first-order kinetics in cells, $\frac{d(R)}{d(t)}$ is determined by $\alpha$ and $R\cdot \beta$.

As steady states had been established, the mRNA level of each gene did not change. We can get:

2 $$\frac{d(R)}{d(t)}=0$$

3 $$R=\frac{\alpha }{\beta }$$

Under the assumption that the labeling efficiency was 100%, all nascent RNA was labeled during the 4sU incubation, and pre-existing RNA would only degrade. So, for nascent RNA (*R*_*n*_), *R*_*n*_ (*t* = 0) = 0 and *α*_*n*_ = *α*. For pre-existing RNA (${R}_{n}$), ${R}_{p}(t\,=\,0)\,=\,R\,=\,\frac{\alpha }{\beta }$ and *α*_*p*_ = 0. Based on these boundary conditions, we could further solve the differential equation above on nascent RNA and pre-existing RNA of each gene.

4 $${R}_{n}=\frac{\alpha \,}{\beta }(1-{e}^{-\beta \times t})$$

5 $${R}_{p}=\frac{\alpha \,}{\beta }{e}^{-\beta \times t}$$

As both $R$ and ${R}_{n}$ were directly measured in *PerturbSci-Kinetics*, and cells were labeled by 4sU for 2 h (*t* = 2), $\beta$ can be calculated from equations 3 and 4. Then, $\alpha$ can be solved by equation 3.

Due to the shallow sequencing and the sparsity of the single-cell expression data, synthesis and degradation rates of DEGs were calculated at the target gene pseudo-cell level. DEGs with only nascent counts or degradation counts were excluded from further examination because their rates could not be estimated.

To examine the significance of synthesis and degradation rate changes upon perturbation, regarding the different cell sizes across different perturbations and NTC, which could affect the robustness of rate calculation, randomization tests were adopted. Only perturbations with cell number ≥50 were examined. For each DEG belonging to each perturbation, background distributions of the synthesis and degradation rate were generated: a subset of cells with the same size as the corresponding perturbed cells was randomly sampled from a mixed pool consisting of corresponding perturbed cells and NTC cells. Then, these cells were aggregated into a background pseudo-cell; synthesis and degradation rates of the gene for testing were calculated as stated above; and the process was repeated for 500 times. Rates = 0 were assigned if only nascent counts or degradation counts were sampled during the process (referred to as invalid samplings), but only genes with fewer than 50 (10%) ‘invalid samplings’ were kept for *P* value calculation. The two-sided empirical *P* values for the synthesis and degradation rate changes were calculated, respectively, by examining the occurrence of extreme values in background distributions compared to the rates from perturbed pseudo-cell. Rate changes with *P* < 0.05 were regarded as significant, and the directions of the rate changes were determined by comparing the rates from the perturbed pseudo-cell with the background mean values.

### Global changes of key statistics upon perturbations

For global synthesis and degradation rate changes, considering the noise from lowly expressed genes, we selected the top 1,000 highly expressed genes from NTC cells and then calculated their synthesis rates and degradation rates in NTC cells and all perturbations with cell number ≥50. Kolmogorov–Smirnov tests were performed to compare rate distributions between each perturbation and NTC cells. The distributions of exonic reads percentage in nascent reads from cells with the same target gene knockdown and NTC cells were compared using Kolmogorov–Smirnov tests to identify genes affecting RNA processing. The proportion of nascent mitochondrial read counts to total mitochondrial read counts was calculated in each single cell, and its distributions between cells with knockdown and NTC cells were compared by Kolmogorov–Smirnov tests to identify the master regulator of mitochondrial mRNA dynamics. In all global statistics examinations, Benjamini–Hochberg multiple hypothesis correction was performed, and comparisons with FDR ≤ 0.05 were considered significant. The medians value from each perturbation and NTC cells were compared to determine the direction of significant changes.

### Coverage analysis

We reprocessed the raw data of AGO2 eCLIP obtained from HeLa cells from Zhang et al.^42^. After adapter trimming, UMI extraction, mapping and UMI-based de-duplication, BAM files were transformed to the single-base coverage by BEDTools^55^. The transcript regions of genes-of-interest were assembled based on the hg38 genome annotation GTF file from GENCODE. In brief, for each gene, the exonic regions were extracted and redivided into 5′ UTR, coding sequence (CDS) and 3′ UTR by the 5′-most start codon and the 3′-most stop codon annotated in the GTF. The AGO2 binding coverages of these designated regions were obtained by intersection and were binned. The gene-specific signal in each bin was normalized by the number of bases in each bin, and the binned coverage of each gene was scaled to be within 0–1. After aggregating scaled coverages of synthesis/degradation-regulated genes, respectively, the lowest point within the CDS was used as the second scaling factor.

Meta-gene coverage analysis was conducted to visualize the gene body distribution of newly transcribed RNA in NTC and AGO2-knockdown samples. Genomic coordinates of protein-coding genes on chromosomes 1–22 and chromosome X were retrieved from the hg38 genome annotation GTF file from GENCODE. Gene bodies were binned into 50 bins, and ordered bins were exported as BED files. For input reads, two nascent read BAM files per group from the pulse-labeling full-coverage SLAM-seq were merged using SAMtools, and then reads with FLAG = 83/163 were assigned to genes on the plus strand, and reads with FLAG = 99/147 were assigned to genes on the minus strand. The gene-specific binned coverages were counted using the BEDTools intersect command. Binned counts of each gene were normalized by total counts in the gene body, and the coverage of any group of genes was finally drawn by averaging the normalized signals across genes.

### Public ChIP-seq, shRNA RNA-seq and GRO-seq data analysis

Genes with detectable expression were identified from shControl/shAGO2 bulk RNA-seq in ENCODE. Processed gene count quantification tables were downloaded from the ENCODE portal. Only genes with mean transcript per million (TPM) > 1 across four samples and with detected expression in at least three of four samples were included. log_2_ FCs of each gene upon AGO2 silencing were calculated by dividing the mean TPM in the shAGO2 group with the mean TPM in the shControl group.

AGO2 ChIP-seq BAM and narrow peak files from ENCODE were merged for identifying TSS binding of AGO2. TSS regions of genes with detectable expression (defined as 4 kb around the TSS) were retrieved, and genes were classified into AGO2 TSS peak^+/−^ genes based on the overlap between their TSS regions with merged AGO2 ChIP-seq narrow peaks. The binding patterns were then visualized using the computeMatrix function in deepTools (3.5.1)^56^.

GRO-seq data were downloaded from the Gene Expression Omnibus (GEO) and reprocessed to depict the transcriptional pausing status of genes. The 3′ end of reads was trimmed against poly(A) by Cutadapt (3.4)^57^, and reads were then aligned to the hg38 reference genome using Bowtie2 (2.3.0)^58^. After filtering out unmapped reads using SAMtools, BAM files were imported to R. TSS proximal regions and transcriptional elongation regions of protein-coding genes with gene lengths ≥1 kb were extracted, and the getPausingIndices() function from the BRGenomics package (3.17)^59^ was used to calculate the pausing indices of genes. Genes detected in both replicates were ranked by the pausing index within the replicate, and an averaged rank was used to study the association with AGO2 TSS binding.

### Reporting summary

Further information on research design is available in the Nature Portfolio Reporting Summary linked to this article.

## Online content

Any methods, additional references, Nature Portfolio reporting summaries, source data, extended data, supplementary information, acknowledgements, peer review information; details of author contributions and competing interests; and statements of data and code availability are available at 10.1038/s41587-023-01948-9.

### Supplementary information

Supplementary Information Supplementary Notes 1–3.

Reporting Summary

Supplementary Tables 1–12 Supplementary Table 1: Primer sequences used in *PerturbSci-Kinetics*. Sequences of the oligo pool library, bulk screen sequencing primer, shortdT RT primers, sgRNA capture primers, ligation primers, sgRNA inner i7 primers and P5/P7 primers were included. The columns indicate the positions on the 96-well plate (Well positions), an identifier of the sequence (Names), the full primer sequence (Sequences) and the barcode sequence (Barcodes). Table 2: Genes and sgRNAs included in the study. Each gene (‘gene_symbol’) has three sgRNAs, and they were named in the format ‘Gene_number’ (‘names’). sgRNA sequences were included in ‘sgRNA_seq’. The ‘gene_class’ is the functional category of each gene. Table 3: Raw sgRNA counts of the bulk screen samples collected at different timepoints. Read counts of each sgRNA (‘sgRNA_name’) from four replicates at day 0 and day 7 were included. Table 4: Relative sgRNA abundance FCs between day 7 and day 0. The ‘Day7_vs_Day0_repX’ is the FC of relative sgRNA abundance at the gene level. Table 5: Information about perturbations that showed significant global synthesis rate changes. Kolmogorov–Smirnov tests and Benjamini–Hochberg multiple hypothesis correction were performed. The ‘adj.p’ is the FDR adjusted for multiple comparisons. The ‘direction’ is the direction of the changes on the global synthesis rate distributions comparing perturbed cells to the NTC cells, and the ‘KD_median/NTC_median’ is the quantitative measurement of the changes. The ‘gene_class’ is the functional category of target genes (‘Perturbations’). Table 6: Information about perturbations that showed significant global degradation rate changes. Kolmogorov–Smirnov tests and Benjamini–Hochberg multiple hypothesis correction were performed. The ‘adj.p’ is the FDR adjusted for multiple comparisons. The ‘direction’ is the direction of the changes on the global degradation rate distributions comparing perturbed cells to the NTC cells, and the ‘KD_median/NTC_median’ is the quantitative measurement of the changes. The ‘gene_class’ is the functional category of target genes (‘Perturbations’). Table 7: Information about perturbations that showed significant nascent exonic reads ratio changes. Kolmogorov–Smirnov tests and Benjamini–Hochberg multiple hypothesis correction were performed. The ‘adj.p’ is the FDR adjusted for multiple comparisons. The ‘direction’ is the direction of the changes on the nascent exonic reads ratio distributions comparing perturbed cells to the NTC cells, and the ‘KD_median/NTC_median’ is the quantitative measurement of the changes. The ‘gene_class’ is the functional category of target genes (‘Perturbations’). Table 8: Information about perturbations that showed significant mitochondrial RNA turnover changes. Kolmogorov–Smirnov tests and Benjamini–Hochberg multiple hypothesis correction were performed. The ‘adj.p’ is the FDR adjusted for multiple comparisons. The ‘direction’ is the direction of the changes in the distributions of mitochondrial nascent/total reads ratio comparing perturbed cells to the NTC cells, and the ‘KD_median/NTC_median’ is the quantitative measurement of the changes. The ‘gene_class’ is the functional category of target genes (‘Perturbations’). Table 9: Steady-state expression and synthesis/degradation dynamics of mitochondrial genes upon *LRPPRC*, *NDUFS2*, *CYC1* and *BCS1L* perturbations. The ‘synth_rate’, ‘synth_FC’, ‘synth_pval’ and ‘synth_direction’ are the synthesis rate of the gene in the perturbed cells, the FC of the synthesis rate of the gene in the perturbed cells compared to the NTC cells, the significance of the synthesis rate change and the direction of the synthesis rate changes. DEG statistical examinations were performed using Monocle2, and the significance of rate changes was examined through randomization tests with empirical two-sided *P* values. The ‘deg_rate’, ‘deg_FC’, ‘deg_pval’ and ‘deg_direction’ are the degradation rate of the gene in the perturbed cells, the FC of the degradation rate of the gene in the perturbed cells compared to the NTC cells, the significance of the degradation rate change and the direction of the degradation rate changes. The ‘DEG_qval’ and ‘DEG_fold.change’ are the multiple comparison-corrected FDR and the FC of the steady-state gene expression change in perturbed cells compared to the NTC cells. Table 10: Filtered DEGs between perturbations with cell number ≥50 and NTC. For each gene (‘Gene_symbol’), the ‘perturbation’ is the target gene in perturbed cells. The ‘DEGs_direction’ is the direction of gene expression changes comparing perturbed cells to the NTC cells, and the ‘DEGs_FC’ is the FC of the gene expression changes comparing perturbed cells to the NTC cells. The ‘max.CPM.between.KD.NTC’ and ‘min.CPM.between.KD.NTC’ are the pseudobulk expression levels of the gene that showed higher and lower expression in perturbed cells or the NTC cells. The expression level was quantified by CPM. The ‘qval’ is the FDR (one-sided likelihood ratio test with adjustment for multiple comparisons). Table 11: DEGs with significant synthesis and/or degradation changes. DEG statistical examinations were performed using Monocle2, and the significance of rate changes was examined through randomization tests with empirical two-sided *P* values. The ‘perturbations’ is the target gene of the perturbed cells, and the ‘Gene_symbols’ is the symbols of DEGs with significant synthesis and/or degradation rate changes in corresponding perturbations. The type of significant rate change of each gene is included in the ‘Regulation_type’. The ‘Synth_deg_FC’, the ‘Synth_deg_direction’ and the ‘Synth_deg_pval’ reflect the FC, the direction of the change and the randomization test *P* value of the rate indicated in the ‘Regulation_type’. ‘DEGs_FC’, ‘DEGs_direction’ and ‘max.expr.between.KD.NTC’ are the FCs of gene expression, the direction of the change and the maximum pseudobulk CPM between the corresponding perturbation and the NTC cells. Table 12: Steady-state expression and synthesis/degradation dynamics of merged DEGs upon *DROSHA* and *DICER1* perturbations. DEG statistical examinations were performed using Monocle2, and the significance of rate changes was examined through randomization tests with empirical two-sided *P* values. The ‘synth_rate’, ‘synth_FC’, ‘synth_pval’ and ‘synth_direction’ are the synthesis rate of the gene in the perturbed cells, the FC of the synthesis rate of the gene in the perturbed cells compared to the NTC cells, the significance of the synthesis rate change and the direction of the synthesis rate changes. The ‘deg_rate’, ‘deg_FC’, ‘deg_pval’ and ‘deg_direction’ are the degradation rate of the gene in the perturbed cells, the FC of the degradation rate of the gene in the perturbed cells compared to the NTC cells, the significance of the degradation rate change and the direction of the degradation rate changes. The ‘DEG_fold.change’ and ‘DEG_qval’ are the FC of the steady-state gene expression change in perturbed cells compared to the NTC cells and the multiple comparison-corrected FDR.

### Source data

Source Data Extended Data Fig. 2 Uncropped agarose gel images for Extended Data Fig. 2c,d-1,d-2,e.

## Data Availability

The data generated by this study can be downloaded in raw and processed forms from the National Center for Biotechnology Information Gene Expression Omnibus (GEO)^60^ (GSE218566). The AGO2 eCLIP data were obtained from the GEO database (GSE115146), and raw data from samples SRR7240709 and SRR7240710 were downloaded. Processed gene counts tables of RNA-seq on shControl/shAGO2 samples were downloaded from the ENCODE portal (ENCSR495YSS and ENCSR898NWE). The AGO2 ChIP-seq BAM and narrow peak files were downloaded from the ENCODE portal (ENCSR151NQL). The GRO-seq data were obtained from the GEO database (GSE97072), and raw data from samples SRR5379790 and SRR5379791 were downloaded. The reference genome hg38 and corresponding genomic annotation GTF file were downloaded from the GENCODE database (release 38, GRCh38.p13). Source data are provided with this paper.

## Code availability

The computation scripts for processing *PerturbSci-Kinetics* are included as supplementary files. Scripts and the user manual are available for open access in GitHub: https://github.com/JunyueCaoLab/PerturbSci_Kinetics (ref. ^61^). Source data are provided with this paper.
