# Supplementary material for: Dissecting key regulators of transcriptome kinetics through scalable single-cell RNA profiling of pooled CRISPR screens
Source: Nat Biotechnol. 2023 Sep 25;42(8):1218–23. doi: 10.1038/s41587-023-01948-9 (PMC10961254; doi:10.1038/s41587-023-01948-9)
Supplement: Supplementary file 2 — Reporting Summary [file 41587_2023_1948_MOESM2_ESM.pdf]

nature research

Corresponding author(s): Junyue Cao

Last updated by author(s): Aug 11, 2023

Reporting Summary

Nature Research wishes to improve the reproducibility of the work that we publish. This form provides structure for consistency and transparency in reporting. For further information on Nature Research policies, see Authors & Referees and the Editorial Policy Checklist.

Statistics

For all statistical analyses, confirm that the following items are present in the figure legend, table legend, main text, or Methods section.

n/a	Confirmed	
☐	☒	The exact sample size (*n*) for each experimental group/condition, given as a discrete number and unit of measurement
☐	☒	A statement on whether measurements were taken from distinct samples or whether the same sample was measured repeatedly
☐	☒	The statistical test(s) used AND whether they are one- or two-sided<br>*Only common tests should be described solely by name; describe more complex techniques in the Methods section.*
☐	☒	A description of all covariates tested
☐	☒	A description of any assumptions or corrections, such as tests of normality and adjustment for multiple comparisons
☐	☒	A full description of the statistical parameters including central tendency (e.g. means) or other basic estimates (e.g. regression coefficient) AND variation (e.g. standard deviation) or associated estimates of uncertainty (e.g. confidence intervals)
☐	☒	For null hypothesis testing, the test statistic (e.g. *F*, *t*, *r*) with confidence intervals, effect sizes, degrees of freedom and *P* value noted<br>*Give P values as exact values whenever suitable.*
☒	☐	For Bayesian analysis, information on the choice of priors and Markov chain Monte Carlo settings
☒	☐	For hierarchical and complex designs, identification of the appropriate level for tests and full reporting of outcomes
☐	☒	Estimates of effect sizes (e.g. Cohen's *d*, Pearson's *r*), indicating how they were calculated

Our web collection on statistics for biologists contains articles on many of the points above.

Software and code

Policy information about availability of computer code

Data collection	No software was used except for Illumina RTA basecalling.
Data analysis	Common, freely available sequencing data analysis software was used to analyze data, as described in Methods: bcl2fastq/v2.19.0.316, python/v2.7.13, trim_galore/v0.6.7, STAR/v 2.7.9a, bedtools/2.30.0, deepTools/3.5.1, R/4.1.1, samtools/1.13, bowtie2/2.3.0, cutadapt/3.4, eulerr/6.1.1, rhdf5/2.38.1, Seurat/4.1.1, clusterProfiler/4.2.2, rtracklayer/1.54.0, GenomicRanges/1.46.1, GenomeInfoDb/1.30.0, IRanges/2.28.0, BiocGenerics/0.40.0, Matrix/1.4-1, dplyr/1.0.10, BRGenomics/3.17.<br><br>Scripts for processing PerturbSci-Kinetics sequencing data was uploaded to github and has been referred in the manuscript.<br><br>The RT-qPCR data was analyzed and visualized using Graphpad Prism/9.2.0. The flow cytometry data was analyzed and visualized using FlowJo/10.8.1.

For manuscripts utilizing custom algorithms or software that are central to the research but not yet described in published literature, software must be made available to editors/reviewers. We strongly encourage code deposition in a community repository (e.g. GitHub). See the Nature Research guidelines for submitting code & software for further information.

Data

Policy information about availability of data

All manuscripts must include a data availability statement. This statement should provide the following information, where applicable:

- Accession codes, unique identifiers, or web links for publicly available datasets
- A list of figures that have associated raw data
- A description of any restrictions on data availability

The data generated by this study can be downloaded in raw and processed forms from the NCBI GEO database (GSE218566). The AGO2 eCLIP data was obtained from the GEO database (GSE115146), and raw data from samples SRR7240709 and SRR7240710 were downloaded. Processed gene counts tables of RNA-seq on

shControl/shAGO2 samples were downloaded from the ENCODE portal (ENCSR495YSS, ENCSR898NWE). The AGO2 ChIP-seq bam and narrow peak files were downloaded from the ENCODE portal (ENCSR151NQL). The GRO-seq data was obtained from the GEO database (GSE97072), and raw data from samples SRR5379790 and SRR5379791 were downloaded. The reference genome hg38 and corresponding genomic annotation gtf file were downloaded from the GENCODE database (Release 38, GRCh38.p13).

Field-specific reporting

Please select the one below that is the best fit for your research. If you are not sure, read the appropriate sections before making your selection.

☒ Life sciences ☐ Behavioural & social sciences ☐ Ecological, evolutionary & environmental sciences

For a reference copy of the document with all sections, see nature.com/documents/nr-reporting-summary-flat.pdf

Life sciences study design

All studies must disclose on these points even when the disclosure is negative.

Sample size	Four independent transductions were performed to examine the reproducibility. For each transduction, during the screening experiment and cell passage, at least 1.4 million cells (~2000x representation for each sgRNA) were kept to minimize the possibility of sgRNA dropout. An average representation of approximately 400 cells for each target gene was chosen when calculating the number of single cells to be profiled.
Data exclusions	No data were excluded from the study.
Replication	The technique was tested and validated in four independent infections in the PerturbSci-Kinetics screen experiment.
Randomization	The screen was conducted in a pooled and massively parallel manner: all sgRNA oligos were randomly cloned into the plasmid, and perturbations took place within the same pool of cells simultaneously after transduction with the pooled lentiviral library. Following screening and metabolic labeling in the same dish, cells received different perturbations were harvested and randomly sampled for PerturbSci-Kinetics library preparation. The entire methodology was designed to minimize the technical batch effects.
Blinding	Investigators were blinded to group allocation during data collection (sequencing) and analysis.

Reporting for specific materials, systems and methods

We require information from authors about some types of materials, experimental systems and methods used in many studies. Here, indicate whether each material, system or method listed is relevant to your study. If you are not sure if a list item applies to your research, read the appropriate section before selecting a response.

Materials & experimental systems

n/a	Involved in the study	
☐	☒	Antibodies
☐	☒	Eukaryotic cell lines
☒	☐	Palaeontology
☒	☐	Animals and other organisms
☒	☐	Human research participants
☒	☐	Clinical data

Methods

n/a	Involved in the study	
☒	☐	ChIP-seq
☐	☒	Flow cytometry
☒	☐	MRI-based neuroimaging

Antibodies

Antibodies used	BV421 Mouse Anti-Human CD221 (BD 565966), BV421 Mouse IgG1 k Isotype Control (BD 562438)
Validation	The antibodies have been validated by the manufacturer. From the manufacturer's website (https://www.bdbiosciences.com/en-us/products/reagents/flow-cytometry-reagents/research-reagents/single-color-antibodies-ruo/bv421-mouse-anti-human-cd221.562593), antibodies mentioned above has been validated by flow cytometric analysis of CD221 expression on human peripheral blood granulocytes.

Eukaryotic cell lines

Policy information about cell lines

Cell line source(s)	HEK293T, HEK293, NIH/3T3, NIH/3T3-CRISPRi, 3T3-L1-CRISPRi, HEK293-idCas9
Authentication	Cell lines were authenticated by checking the morphology and mapping sequencing reads from different cell lines to species-specific reference genomes.

Mycoplasma contamination	Cell lines were not tested for Mycoplasma contamination.
Commonly misidentified lines (See ICLAC register)	No commonly misidentified cell lines were used.

Flow Cytometry

Plots

Confirm that:

☒ The axis labels state the marker and fluorochrome used (e.g. CD4-FITC).

☒ The axis scales are clearly visible. Include numbers along axes only for bottom left plot of group (a 'group' is an analysis of identical markers).

☒ All plots are contour plots with outliers or pseudocolor plots.

☒ A numerical value for number of cells or percentage (with statistics) is provided.

Methodology

Sample preparation	For flow cytometry validation, 1e6 cells of each sample were harvested and resuspended in 100ul of PBS-0.1% sodium azide-2% FBS. BV421 Mouse Anti-Human CD221 (BD 565966) and BV421 Mouse IgG1 k Isotype Control (BD 562438) at the final concentration of 10 ug/ml were added, and reactions were incubated at 4 °C in the dark with rotation for 30 minutes. Cells were then washed twice using PBS-0.1% sodium azide-2% FBS, and fluorescence signals were recorded.
Instrument	BD Aria III
Software	FlowJo
Cell population abundance	No specific cell sub-type was selected. Flowcytometry was used for membrane protein level measurement only.
Gating strategy	FSC-A and FSC-W were firstly used to remove debris, and SSC-A and SSC-W were then used to further remove low quality cells and doublets/multiplets. For sgRNA transduced cells (GFP+), 488C-515-A+ cells were selected. For dCas9 induced cells (mCherry +), 561C-610-A+ cells were selected. A representative gating strategy is shown in Extended Data Figure 4b.

☒ Tick this box to confirm that a figure exemplifying the gating strategy is provided in the Supplementary Information.
