# Supplementary figures and images for: Dissecting key regulators of transcriptome kinetics through scalable single-cell RNA profiling of pooled CRISPR screens
Source: Nat Biotechnol. 2023 Sep 25;42(8):1218–23. doi: 10.1038/s41587-023-01948-9 (PMC10961254; doi:10.1038/s41587-023-01948-9)

# Agarose 2%

M 1 2 3 4 5 6 7 8 9 10

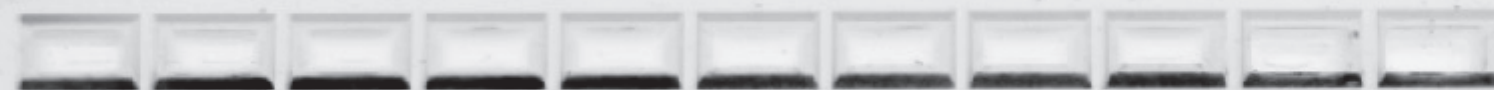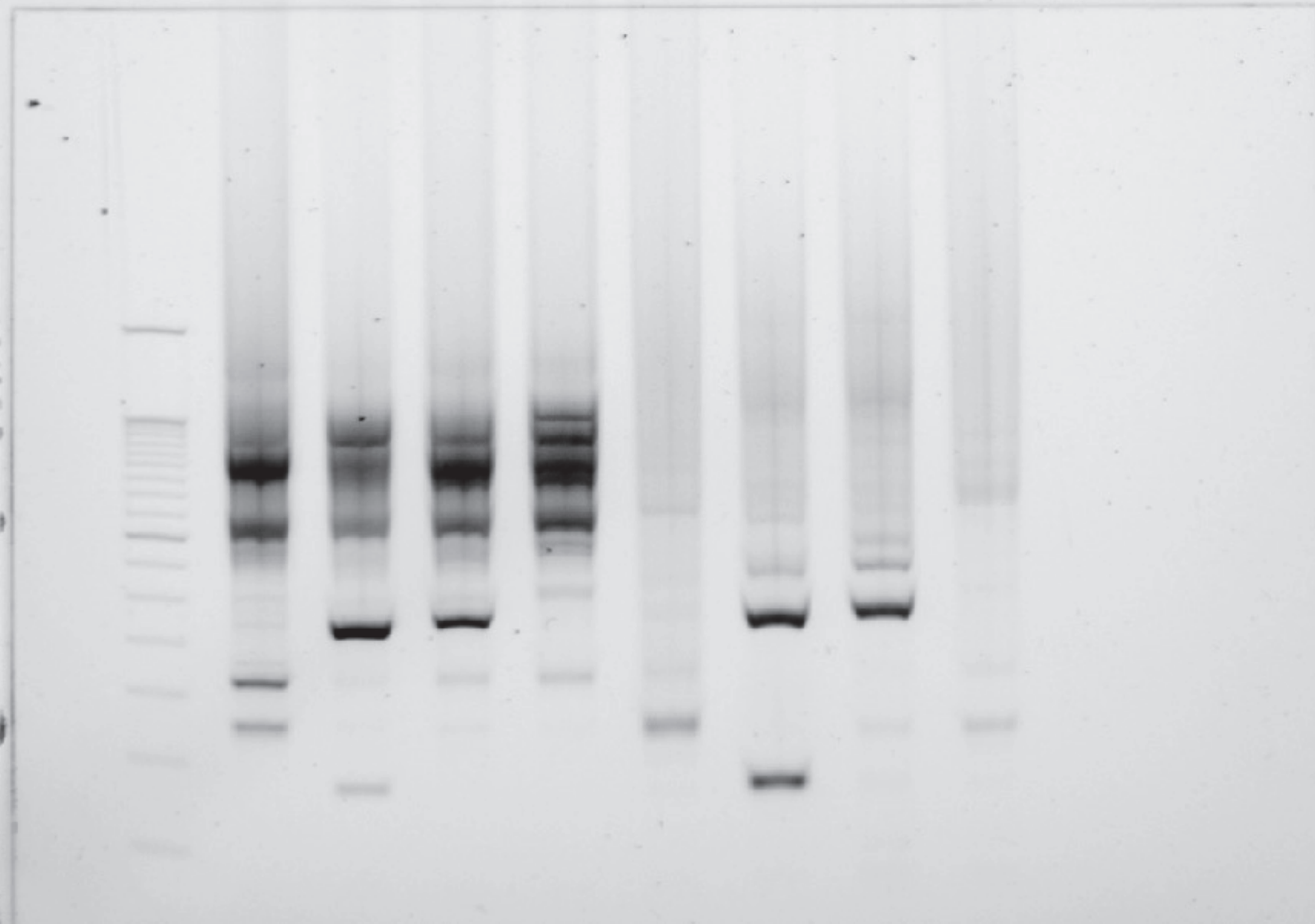

2d-1

**Agarose 2%**

M 1 2 3 4 5 6 7 8 9 10

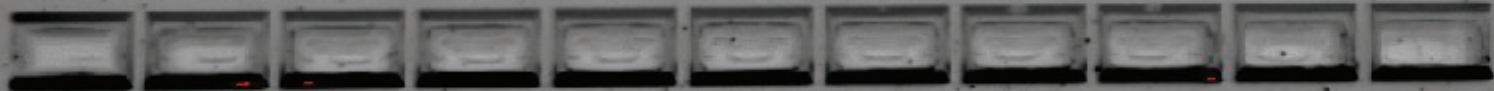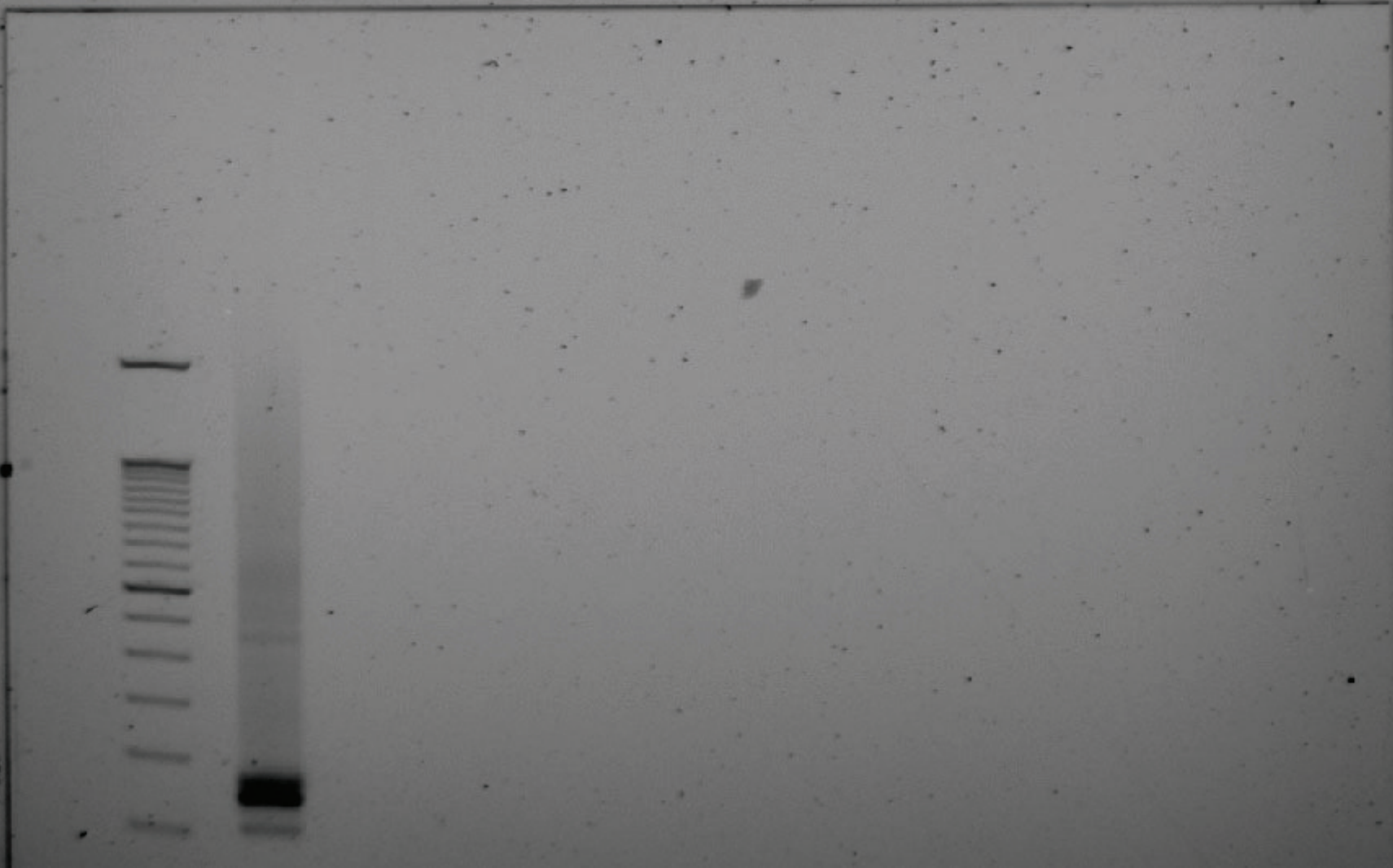

2d-2

# Agarose 2%

M 1 2 3 4 5 6 7 8 9 10

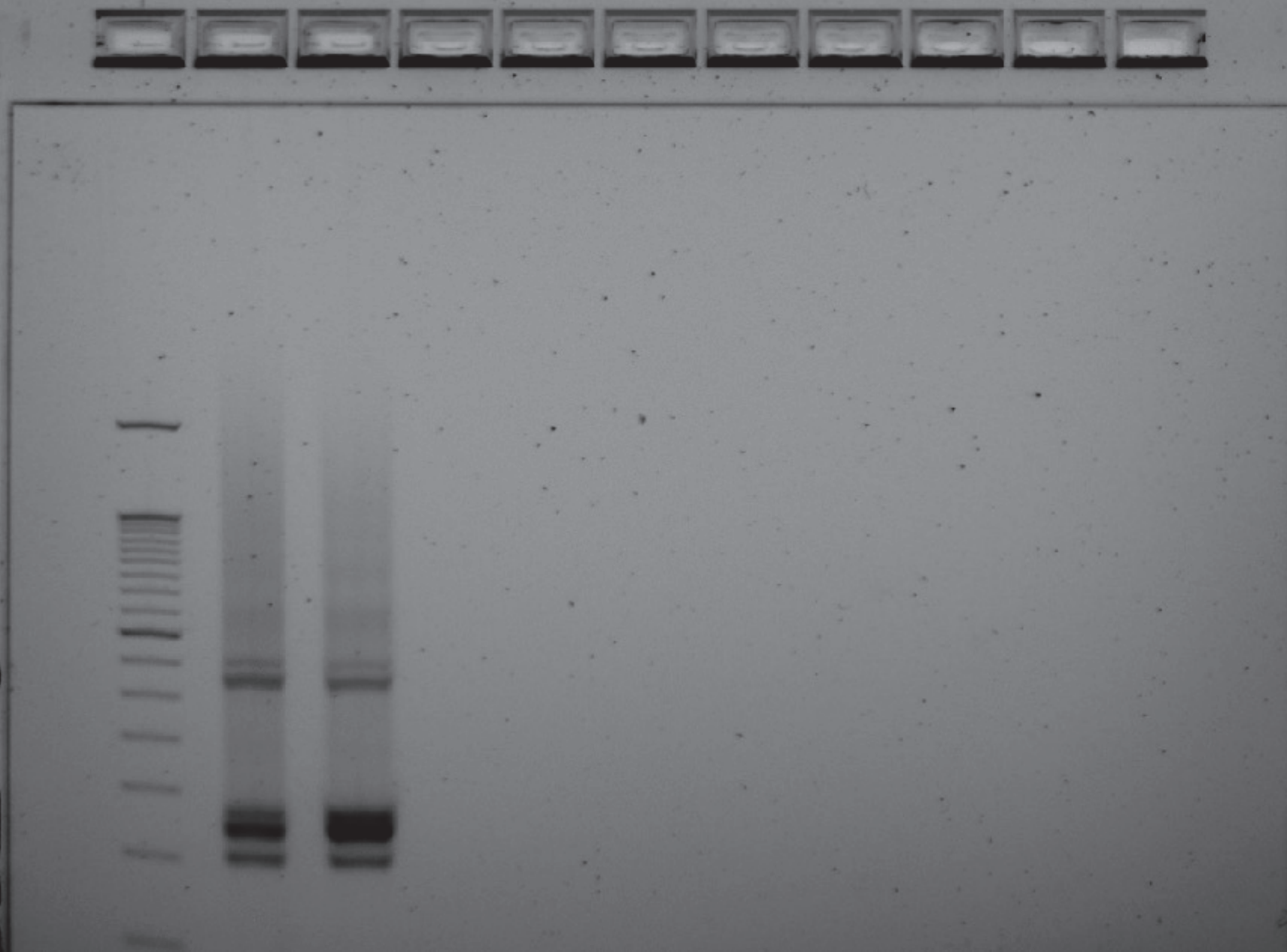

# Agarose 2%

M 1 2 3 4 5 6 7 8 9 10

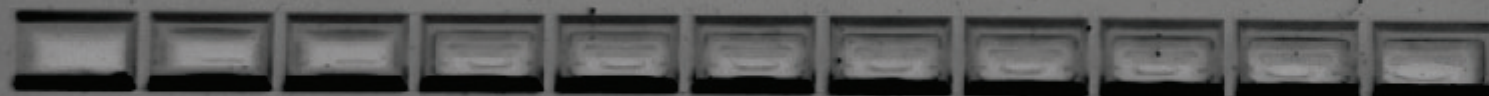

Supplement: Supplementary file 4 — Uncropped agarose gel images for Extended Data Fig. 2c,d-1,d-2,e. [file 41587_2023_1948_MOESM4_ESM.pdf]
